# Turning *Saccharomyces cerevisiae* into a Frataxin-Independent Organism

**DOI:** 10.1371/journal.pgen.1005135

**Published:** 2015-05-21

**Authors:** Heeyong Yoon, Simon A. B. Knight, Alok Pandey, Jayashree Pain, Serdar Turkarslan, Debkumar Pain, Andrew Dancis

**Affiliations:** 1 Department of Medicine, Division of Hematology-Oncology, Perelman School of Medicine, University of Pennsylvania, Philadelphia, Pennsylvania, United States of America; 2 Department of Pharmacology and Physiology, New Jersey Medical School, Rutgers University, Newark, New Jersey, United States of America; 3 Institute for Systems Biology, Seattle, Washington, United States of America; Universidad de Sevilla, Spain

## Abstract

Frataxin (Yfh1 in yeast) is a conserved protein and deficiency leads to the neurodegenerative disease Friedreich’s ataxia. Frataxin is a critical protein for Fe-S cluster assembly in mitochondria, interacting with other components of the Fe-S cluster machinery, including cysteine desulfurase Nfs1, Isd11 and the Isu1 scaffold protein. Yeast Isu1 with the methionine to isoleucine substitution (M141I), in which the *E*. *coli* amino acid is inserted at this position, corrected most of the phenotypes that result from lack of Yfh1 in yeast. This suppressor Isu1 behaved as a genetic dominant. Furthermore frataxin-bypass activity required a completely functional Nfs1 and correlated with the presence of efficient scaffold function. A screen of random Isu1 mutations for frataxin-bypass activity identified only M141 substitutions, including Ile, Cys, Leu, or Val. In each case, mitochondrial Nfs1 persulfide formation was enhanced, and mitochondrial Fe-S cluster assembly was improved in the absence of frataxin. Direct targeting of the entire *E*. *coli* IscU to *∆yfh1* mitochondria also ameliorated the mutant phenotypes. In contrast, expression of IscU with the reverse substitution i.e. IscU with Ile to Met change led to worsening of the *∆yfh1* phenotypes, including severely compromised growth, increased sensitivity to oxygen, deficiency in Fe-S clusters and heme, and impaired iron homeostasis. A bioinformatic survey of eukaryotic Isu1/prokaryotic IscU database entries sorted on the amino acid utilized at the M141 position identified unique groupings, with virtually all of the eukaryotic scaffolds using Met, and the preponderance of prokaryotic scaffolds using other amino acids. The frataxin-bypassing amino acids Cys, Ile, Leu, or Val, were found predominantly in prokaryotes. This amino acid position 141 is unique in Isu1, and the frataxin-bypass effect likely mimics a conserved and ancient feature of the prokaryotic Fe-S cluster assembly machinery.

## Author Summary

Frataxin was discovered because mutations in the corresponding gene cause the neurodegenerative disease Friedreich’s ataxia. The finding that frataxin protein physically associates with scaffold proteins Isu1/IscU places it squarely in the pathway of Fe-S cluster assembly. Fe-S clusters are essential cofactors for many proteins involved in cellular respiration, DNA repair, translation and other processes. Frataxin is conserved throughout evolution, being present in eukaryotes such as yeast and human and in some prokaryotes including *E*. *coli*. However, differences exist between the eukaryotic and prokaryotic forms of frataxin. The eukaryotic forms are critical for Fe-S cluster assembly whereas prokaryotic forms are more dispensable. We found that a key to this difference is a single amino acid in the scaffold protein Isu1 at position 141. Changes of the eukaryotic amino acid, Met, to prokaryotic amino acids, Ile, Leu, Cys, or Val, rendered mitochondria more frataxin-independent. No other changes were able to replicate this effect. Thus, Isu1 containing Met at position 141 may have coevolved with frataxin in eukaryotes, conferring frataxin-dependence. In contrast, the appearance of other amino acids at this position may have rendered prokaryotic cells less dependent on frataxin.

## Introduction

Frataxin is a highly conserved protein that is found in both prokaryotic and eukaryotic organisms [1]. The protein was originally identified based on its connection to Friedreich’s ataxia, which is an inherited neurodegenerative and cardiodegenerative disease resulting from a deficiency of frataxin [2]. Recently a mitochondrial Fe-S cluster assembly protein complex was identified consisting of frataxin in association with the cysteine desulfurase Nfs1, the small eukaryote-specific protein Isd11, and the scaffold protein Isu1 [3,4,5]. This protein complex serves to synthesize Fe-S cluster intermediates on Isu1 for subsequent transfer to the myriad proteins that use Fe-S cluster cofactors [6,7]. Iron-sulfur cluster intermediates contain iron and sulfur bound to the Isu1 scaffold in a [Fe_2_S_2_] configuration [8]. Although the precise function of frataxin has not been defined, it probably plays a role in sulfur and/or iron donation to Fe-S cluster intermediates [9,10].

The entire process of Fe-S cluster biogenesis is highly conserved between eukaryotic mitochondria and prokaryotic organisms [6,7]. Similar components are present, and in many cases the corresponding proteins function as orthologs. Sulfur for Fe-S cluster synthesis is derived from cysteine via the action of a cysteine desulfurase (Nfs1 in yeast, IscS in bacteria). This enzyme binds the amino acid cysteine in a substrate-binding site via the pyridoxal phosphate (PLP) cofactor [8]. The bound substrate is subjected to nucleophilic attack by an active site cysteine present on a moveable loop of the protein, forming a persulfide (e.g. Nfs1-S-SH in yeast). The persulfide sulfur is then transferred to recipients including Isu1 and used in building Fe-S clusters [11]. Frataxin (Yfh1 in yeast, CyaY in *E*. *coli*) interacts with the cysteine desulfurase and may be involved in regulating the enzyme activity. Whereas a positive regulatory effect has been observed for the yeast or human proteins [12,13], a negative regulatory effect has been observed for the *E*. *coli* homolog [14], and this difference has still not been explained [15]. Isd11 is a small accessory subunit that interacts with the eukaryotic Nfs1 and is necessary for its cysteine desulfurase activity [16]. However, Isd11 is eukaryote specific, being entirely absent from prokaryotic lineages [17]. Iron combines with sulfur on the scaffold protein to form Fe-S cluster intermediates. The scaffolds (Isu1 in yeast, IscU in *E*. *coli*) are highly homologous proteins [18]. The iron donation step is poorly characterized, and frataxin has also been implicated in this step. Both yeast and *E*. *coli* frataxins bind iron with low affinity on acidic residues *in vitro* and interact with their respective scaffold proteins *in vitro* and *in vivo*, and thus they may participate in iron donation [19,20]. Electrons provided by ferredoxin (Yah1 in yeast, Fdx in *E*. *coli*) are needed for reduction of iron or sulfur during Fe-S cluster intermediate formation [21]. Following formation of the intermediate on Isu1 or IscU, coordinated by three critical cysteines in the protein backbone, Hsp70 chaperones and cochaperones (Ssq1 and Jac1 in yeast, HscA and HscB in *E*. *coli*) interact with the scaffolds, mediating Fe-S cluster transfer in an ATP-dependent manner [22].

In terms of phenotypes resulting from frataxin deficiency, however, eukaryotes and prokaryotes show major differences. Total lack of frataxin is lethal in humans and metazoans [4]. Deletion of the *YFH1* gene in yeast is associated with extremely deleterious effects, including slow growth, oxidant sensitivity, heme deficiency and lack of Fe-S clusters [23,24]. In addition, frataxin deficiency is associated with a curious iron homeostatic phenotype characterized by constitutive and unregulated cellular iron uptake. Within the cell iron accumulates in mitochondria in the form of biologically unavailable ferric phosphate nanoparticles. This constellation of findings apparently results from defective Fe-S proteins in the iron-sensing machinery [25,26]. In contrast to the yeast mutants, the effects of frataxin deletion in *E*. *coli* are mild. The bacterial deletion strain shows normal growth and does not exhibit iron homeostatic abnormalities or sensitivity to oxidative stress, although in one report the protein level for respiratory complex I was reduced [27].

A spontaneously occurring mutation in a frataxin-deleted yeast strain was found to effectively bypass the severe *Δyfh1* phenotypes, restoring normal growth, Fe-S cluster protein levels, iron homeostasis, heme synthesis, and oxidative stress resistance. The effect was conferred by the Met to Ile change of amino acid 141 in the scaffold protein Isu1 [28]. The altered Isu1 was able to bind and activate the Nfs1 cysteine desulfurase in the absence of frataxin, thus providing a possible explanation for the bypass activity [29,30]. Interestingly, isoleucine is the amino acid utilized by *E*. *coli* in the homologous position of IscU. Thus in yeast lacking frataxin, the Met to Ile change in Isu1, by substituting the *E*. *coli* amino acid at this position, effectively rendered yeast more frataxin independent and more "prokaryote like".

Here we have delved more into the genetics of this frataxin-bypass phenomenon, finding more prokaryotic features of Isu1 bypass mutants. Randomly selected Isu1 bypass mutants were confined to a single amino acid position, and the amino acids conferring bypass were all present in homologous prokaryotic proteins. The prokaryotic homologs were identified both in organisms with frataxin and in organisms without frataxin, underscoring the frataxin-independence associated with these particular scaffold mutants. The entire *E*. *coli* IscU (targeted to mitochondria with a leader sequence) conferred more frataxin-independence, whereas a reverse substitution, in which the eukaryotic amino acid Met was introduced at the same position, conferred more frataxin-dependence. Examination of the set of Isu1/IscU sequences in available databases also suggests that Isu1-Met appears almost exclusively in eukaryotes and likely coevolved with frataxin. The substituted Isu1-Ile probably mimics a conserved and ancient feature of the prokaryotic Fe-S cluster assembly complex, relieving the frataxin requirement.

## Results

### Genetic dominance of the M141I Isu1 or Isu2 substitution conferring frataxin-bypass

The frataxin-bypass mutant was isolated as a spontaneously arising clone of more rapidly growing cells in a background of a frataxin-deleted haploid yeast strain (*Δyfh1*) [28]. The bypass activity was conferred by a single point mutation (ATG to ATA), changing the amino acid of codon 141 of *ISU1* from Met to Ile. However yeast *S*. *cerevisiae* carries two redundant genes coding for Fe-S cluster scaffolds, *ISU1* and *ISU2* [31]. The initial presentation of the suppressor or bypass mutant suggested that it was genetically dominant, because it was able to bypass *Δyfh1* even in the presence of a normal copy of *ISU2*.

**Fig 1:**
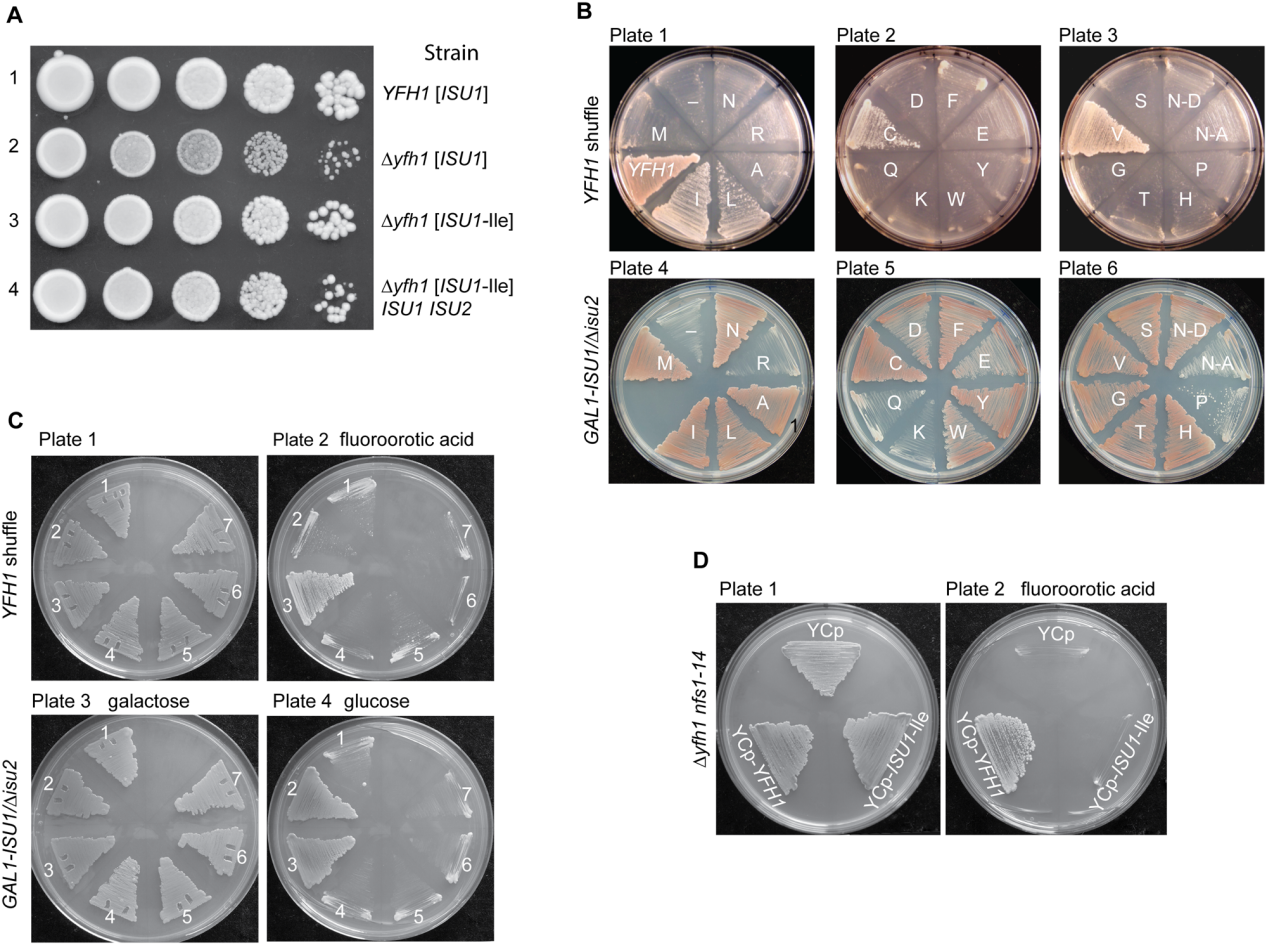
Genetic manipulations of *ISU1*-Ile and other *ISU1* alleles. (A) Genetic dominance of *ISU1*-Ile conferring frataxin-bypass. Strains *YFH1* [*ISU1*], Δ*yfh1* [*ISU1*], Δ*yfh1* [*ISU1*-Ile], and Δ*yfh1* [*ISU1*-Ile] *ISU1 ISU2* (Table 1) were compared by spotting serial 5-fold dilutions of 10^5^ cells on YPAD plates and photographing three days later. (B) Frataxin-bypass function and scaffold function of *ISU1* alleles. A series of plasmids was constructed in the YCplac22 backbone. These plasmids carried native *ISU1* or *ISU1* alleles with substitutions of residue M141 to all 19 other standard amino acids. N123D or N123A substitutions were also constructed, predicted to give disordered or structured conformations, respectively [34]. Plasmid-borne *YFH1* and empty vector (-) were included as controls. To test frataxin-bypass function the *ISU1* alleles were transformed into the *YFH1* shuffle strain, and the transformed cells were transferred to fluoroorotic acid (FOA) plates to remove the covering *YFH1-URA3* plasmid and expose the *Δyfh1* phenotype (plates 1–3). To test scaffold function the *ISU1* alleles were transformed into the *GAL1-ISU1/Δisu2* strain and plated on raffinose carbon source to repress genomic *GAL1-ISU1*. (C) Effects of cysteine mutations. Plasmids with various cysteine substitutions in Isu1 were tested for frataxin-bypass function by introducing them into the *YFH1* shuffle strain and counterselecting on FOA (plates 1 and 2). The Isu1 substitutions were also tested for scaffold function by introducing them into the *GAL1-ISU1/Δisu2* strain and shifting the carbon source from galactose to glucose (plates 3 and 4). Key to transformants. 1. Empty plasmid YCplac22, 2. *ISU1*, 3. *ISU1*-M141I, 4. *ISU1*-C69A-M141I, 5. *ISU1*-C96A-M141I, 6. *ISU1*-C139A-M141I, 7. *ISU1*-C139A-M141C. (D) *Δyfh1 nfs1-14* double mutant does not support frataxin-bypass by *ISU1*-M141I. Shuffle strain 113–26 (*Δyfh1 nfs1-14* [pRS416-*YFH1*]) was transformed with YCplac22, YCplac22-*YFH1*, or YCplac22-*ISU1*-Ile. Transformants were patched onto tryptophan drop-out medium without (plate 1) or with (plate 2) FOA.

In order to evaluate this genetic dominance further, matched *Δyfh1* strains were compared, one expressing a single copy of substituted *ISU1* and deleted *ISU2*, called *Δyfh1* [*ISU1*-Ile] (Table 1) and another expressing both *ISU1* and *ISU2* in addition to the plasmid-borne substituted Isu1-M141I, called *Δyfh1* [*ISU1*-Ile] *ISU1 ISU2* (Table 1). As assessed by colony formation, both strains showed improved growth compared with the *Δyfh1* control (Fig 1A, compare rows 3 and 4 to row 2), although the single copy [*ISU1*-Ile] showed slightly better *Δyfh1* suppression activity as assessed by growth (Fig 1A, compare rows 3 and 4), and other phenotypes such as iron uptake and Fe-S cluster levels. Thus, a high degree of genetic dominance of the Isu1 Met to Ile substitution was observed in producing reversal of *Δyfh1* phenotypes.

**Table 1:** List of yeast strains.

Strain number	Name	Genotype	Source
70–31	*YFH1* shuffle	*MATa ura3-52 lys2-801(amber) ade2-101(ochre) trp1-Δ63 his3-Δ200 leu2-Δ1 Δyfh1*::*HIS3 ISU1 ISU2* [*pGAL1*-*YFH1*, *URA3*]	[28]
109–9	*YFH1* (*Δisu1*) shuffle	*MATa ura3-52 lys2-801(amber) ade2-101(ochre) cyh2 Δyfh1*::*TRP1 Δisu1*::*HIS3MX6 ISU2* [pRS318- *YFH1*-*CYH2*-*LEU2*]	This work
110–66		*MATa ura3-52 lys2-801(amber) ade2-101(ochre) trp1-Δ63 his3-Δ200 leu2-Δ1 Δyfh1*::*HIS3 ISU1 ISU2* [*pGAL1-YFH1-URA3*][YCplac22-*ISU1-*M141I]	[28]
108–54	*Δyfh1 ISU1 ISU2* [*ISU1*-Ile]	*MATa ura3-52 lys2-801(amber) ade2-101(ochre) trp1-Δ63 his3-Δ200 leu2-Δ1 Δyfh1*::*HIS3 ISU1 ISU2* [YCplac22-*ISU1*-M141I]	This work
113–26		*MAT*? *ura3-52 lys2-801(amber) ade2-101(ochre) trp1-Δ63 leu2-Δ1 cyh2 nfs1-14*::*LEU2 Δyfh1*::*HIS3 ISU1 ISU2* [pRS416-*YFH1*]	This work
115–10	*GAL1-ISU1/Δisu2* (Parent)	*MATa lys2-801(amber) ade2-101(ochre) trp1-Δ63 leu2-Δ1 YFH1 Δisu2*::*URA3 His3MX6*:*GAL1-ISU1*	This work
115–26	Δ*yfh1* [*ISU1*]	Parent with [YCplac22-*ISU1*] *Δyfh1*::*LEU2*	[30]
115–28	Δ*yfh1* [*ISU1*-Ile]	Parent with [YCplac22-*ISU1*-M141I] *Δyfh1*::*LEU2*	[30]
116–51	Δ*yfh1* [*ISU1*-Val]	Parent with [YCplac22-*ISU1*-M141V] *Δyfh1*::*LEU2*	This work
116–53	Δ*yfh1* [*ISU1*-Leu]	Parent with [YCplac22-*ISU1*-M141L] *Δyfh1*::*LEU2*	This work
116–54	Δ*yfh1* [*ISU1*-Cys]	Parent with [YCplac22-*ISU1*-M141C] *Δyfh1*::*LEU2*	This work
117–36	*YFH1* [*iscU*]	Parent with [YCplac22-*iscU E*. *coli*]	This work
117–37	*YFH1* [*iscU*-Met]	Parent with [YCplac22-*iscU*-I108M *E*. *coli*]	This work
117–40 117–42	Δ*yfh1* [*iscU*]	Parent with *Δyfh1*::*LEU2* [YCplac22-*iscU E*.*coli*]	This work
117–44 117–46	Δ*yfh1* [*iscU*-Met]	Parent with *Δyfh1*::*LEU2* [YCplac22-*iscU*-I108M *E*. *coli*]	This work
114–58	*YFH1* [*ISU1*]	Parent with [YCplac22-*ISU1*]	This work

*ISU1* and *ISU2* encode highly homologous proteins, with 83% amino acid identity. Isu1 and Isu2 proteins are functionally redundant, although the endogenous expression level of Isu1 is roughly seven times higher than that of Isu2, and Isu1 represents most of the cellular Isu protein in a wild-type strain [32]. Significantly the critical Met (amino acid 141 in Isu1 or 133 in Isu2) is present in both proteins. The adjacent cysteine that functions as an Fe-S cluster ligand and the adjoining frataxin-binding motif are also present in both proteins [33]. *ISU2* and *ISU2*-Ile, in which the Met-133 was changed to Ile, were experimentally evaluated. Results show that *ISU2*-Ile conferred frataxin-bypass activity in a *YFH1* shuffle strain (S1 Fig). Bypass activity was conferred whether it was expressed from the native *ISU2* promoter or from the *ISU1* promoter (S1 Fig). Thus, genetic dominance for frataxin-bypass by *ISU2*-Ile was observed, similar to that of *ISU1*-Ile. Furthermore, the *ISU2* and *ISU2*-Ile constructs were also able to support growth of the *GAL1-ISU1/Δisu2* strain indicating that they were functional as Isu. The reason that only the *ISU1*-Ile was isolated and *ISU2*-Ile was not isolated in the original screen for *Δyfh1* suppressors is probably due to the lack of saturation of this genetic screen.

### Isu1 substitutions at M141: Frataxin-bypass function and scaffold function

All 20 possible amino acids were substituted at position M141 in a single copy plasmid-borne *ISU1*. Each mutant form of *ISU1* was tested for frataxin-bypassing activity by transforming the plasmids into a *YFH1* shuffle strain, followed by counterselection with fluoroorotic acid on raffinose medium to remove the covering plasmid (Fig 1B, plates 1–3). After counterselection, robust growth was noted for positive control YCplac22-*YFH1*, and slow growth was noted for the empty plasmid, YCplac22 (Fig 1B, plate 1, “*YFH1*” versus “-”), consistent with the important role of frataxin for normal growth. The *ISU1*-Ile plasmid with the Met to Ile change restored robust growth in the absence of *YFH1* (Fig 1B, plate 1, “I”). Similarly, plasmids substituted at the same amino acid position and containing *ISU1*-Leu, *ISU1*-Cys, and *ISU1*-Val, conferred improved growth (Fig 1B, plates 1–3). These *ISU1* alleles thus carried frataxin-bypass activity and were genetically dominant, given that wild-type genomic copies of *ISU1* and *ISU2* were still present.

Fe-S cluster assembly scaffold function was tested separately. The collection of *ISU1* alleles was transformed into the *GAL1-ISU1/Δisu2* strain. In this strain the redundant *ISU2* was deleted and *ISU1* was placed under control of *GAL1*, a galactose-dependent promoter. When the transformants were shifted to a non-inducing carbon source (i.e. raffinose-based medium), only plasmid-borne *ISU1* was expressed, allowing scaffold function for each allele to be scored. The wild-type *ISU1* (Fig 1B, plate 4, M) supported normal growth, and a large set of *ISU1* plasmids with amino acid substitutions also supported normal growth, indicating that these were highly functional *ISU1* proteins (Fig 1B, plates 4–6, I, L, A, N, C, F, Y, S, V, G, T and H). Importantly, all of the substitutions conferring frataxin-bypass activity (Fig 1B, plates 1–3, I, L, C, and V) were also functional *ISU1* scaffold proteins. Another set of substitutions was partially functional as shown by slowed growth (Fig 1B, plate 5, D, E and W), and these mutants tended to accumulate iron, indicating that they were hypomorphic mutants in the Fe-S cluster assembly pathway. A small set of substituted alleles was completely non-functional and did not grow at all on the raffinose plates (Fig 1B, plates 4–6, R, Q, K and P).

The *E*. *coli* ortholog, IscU, was studied by NMR methodology [34] and found to assume interconverting disordered or structured conformations. When a conserved amino acid in the primary sequence, Asn 123 in the Isu1 numbering, was substituted with Asp, the conformation of the mutant protein was shown to be shifted preferentially to the disordered form. Alternatively when Asn 123 was substituted with Ala, the conformation was shifted to the structured form [34]. We wondered if the frataxin-bypassing activity could be related to one or the other of these conformations. The Asp change (N-D; presumably disordered) and the Ala change (N-A; presumably structured) were introduced into YCplac22-*ISU1* and the respective plasmids were transformed into the *YFH1* shuffle strain. However, neither conferred any bypass activity (Fig 1B, plate 3, N-D and N-A). The Asp 123 form conferred growth to the *GAL1-ISU1/Δisu2* strain indicating efficient scaffold function, but the Ala 123 form was poorly functional in this assay (Fig 1B, plate 6, N-D versus N-A).

### Substitutions of critical cysteine residues inactivate both frataxin-bypass activity and scaffold activity of *ISU1*-Ile

Isu1 with the Met to Ile substitution, *ISU1-*Ile, exhibited both frataxin-bypassing activity and scaffold activity. Therefore the question arose of whether both activities must necessarily reside in the same molecule. Alternatively, the *ISU1*-Ile might act on wild-type *ISU1* protein, stimulating it to perform as the primary scaffold. The existence of a protein complex containing the suppressor *ISU1*-Ile protein and the wild-type *ISU1* protein could provide a physical context for such stimulation to occur [35,36]. To begin to address this question genetically, the three cysteine residues of Isu1, presumed to be Fe-S cluster ligands [31], were individually replaced by alanine. The C69A, C96A and C139A forms of Isu1 were introduced into strain *GAL1-ISU1/Δisu2*, and they were unable to support growth on their own in non-inducing glucose-containing medium. Double mutants were then constructed in which the critical cysteine substitutions were combined with the suppressor *ISU1*-Ile change of residue 141 in the same molecule. The mutated *ISU1* alleles, C69A-M141I, C96A-M141I and C139A-M141I, were tested for frataxin-bypass activity in the *YFH1* shuffle strain, and no bypass activity was observed (Fig 1C, plate 2). Likewise the mutated *ISU1* alleles were tested for scaffold activity by expression in the *GAL1-ISU1/Δisu2* strain in glucose, and no complementation was observed (Fig 1C, plate 4). The frataxin-bypass activity of the Cys substitution of residue 141 raised the possibility that the cysteine at this location was functioning as an alternative Fe-S cluster ligand instead of Cys 139. In a genetic experiment, Cys 139 was replaced with alanine in the presence of the M141C substitution. However, in this case, neither scaffold activity nor bypass activity was observed (Fig 1C, plates 2 and 4, number 7), making it unlikely that the Cys substituted at position 141 can function as an alternative Fe-S cluster ligand. In summary, substitutions that abrogated Isu1 scaffold function by altering the Fe-S cluster ligands also abrogated frataxin-bypass function.

### Cysteine desulfurase hypomorphic allele *nfs1-14* does not permit frataxin-bypass by *ISU1*-Ile

A mutant allele of *NFS1*, *nfs1-14*, was identified because of its effects on iron homeostasis, and it was later found to be associated with decreased cysteine desulfurase activity in mitochondria [16]. The cells carrying this mutant allele were viable and grew well in most media despite the low activity of cysteine desulfurase. However, the combination of *nfs1-14* and *Δyfh1* was synthetically lethal (Fig 1D, YCp). *YFH1* (Fig 1D, YCp-*YFH1*) introduced into the *nfs1-14 Δyfh1* strain restored growth. However, although *ISU1*-Ile was able to bypass *Δyfh1* alone, it was unable to rescue the *nfs1-14 Δyfh1* double mutant (Fig 1D, YCp-*ISU1*-Ile). These data suggest that a threshold level of cysteine desulfurase activity is required for the frataxin-bypass activity of *ISU1*-Ile. Recent biochemical data indicate that frataxin stimulates the cysteine desulfurase activity of Nfs1 [13]. Furthermore, the suppressor *ISU1*-Ile acts in a similar fashion to stimulate Nfs1 even in the absence of frataxin, perhaps explaining its bypass activity [29]. Thus it makes sense that in the absence of a sufficiently active Nfs1, bypass of *Δyfh1* by the Ile-substituted *ISU1* will not occur.

### Biochemical characteristics of newly discovered frataxin-bypassing mutant alleles of *ISU1*

**Fig 2:**
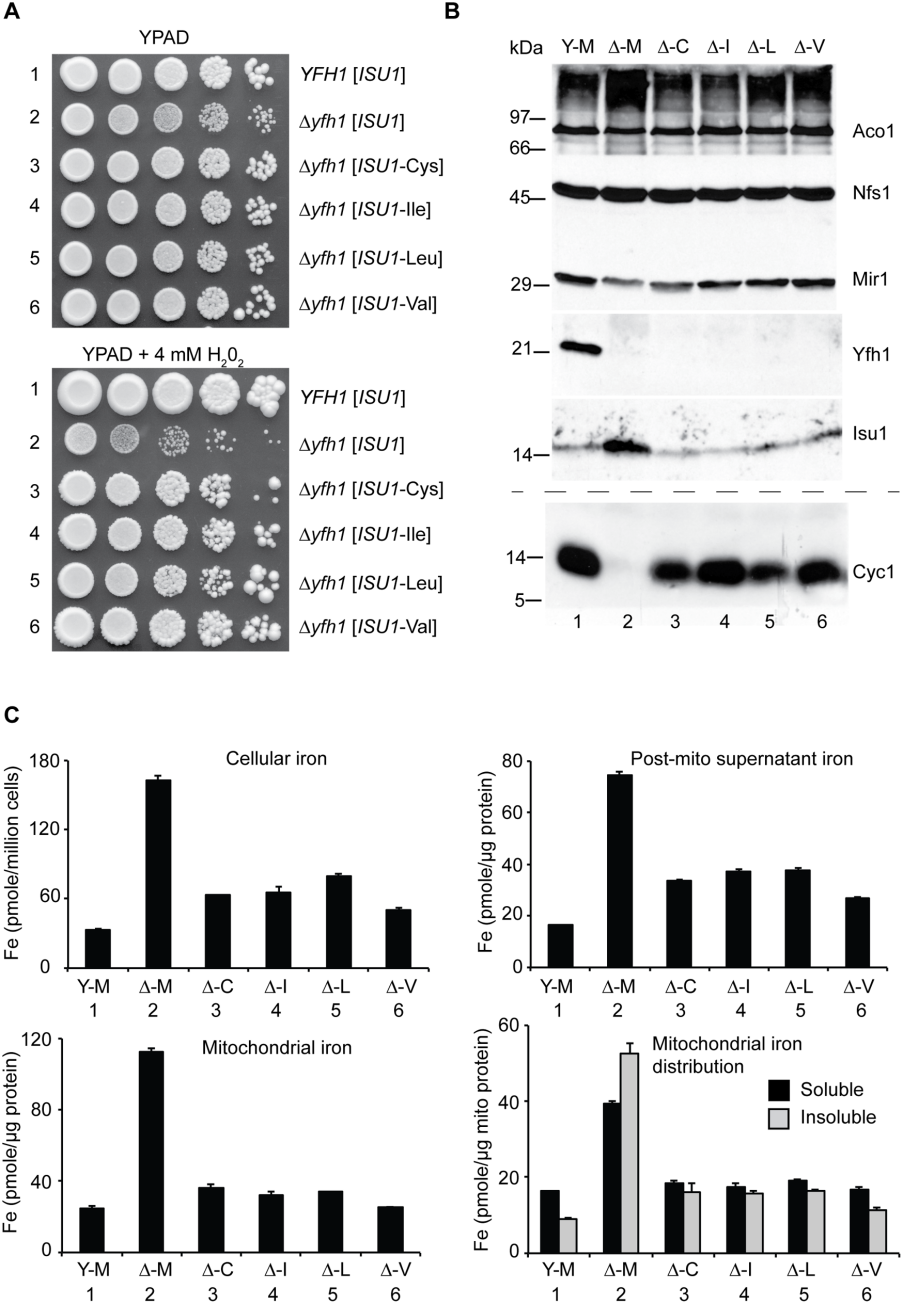
Characterization and comparison of frataxin-bypass substitutions of *ISU1* amino acid 141: *ISU1*-Cys, *ISU1*-Ile, *ISU1*-Leu and *ISU1*-Val. (A) Growth defects in *Δyfh1* are corrected by the frataxin-bypass suppressors. The strains *YFH1* [*ISU1*] (Y-M), Δ*yfh1* [*ISU1*] (Δ-M), and suppressor strains Δ*yfh1* [*ISU1*-Cys] (Δ-C), Δ*yfh1* [*ISU1*-Ile] (Δ-I), Δ*yfh1* [*ISU1*-Leu] (Δ-L), and Δ*yfh1* [*ISU1*-Val] (Δ-V), were grown in an argon-filled chamber, and serial dilutions were spotted on YPAD agar, without or with 4 mM hydrogen peroxide. (B) Mitochondrial protein levels by immunoblotting. Strains were inoculated from the argon-filled chamber into aerobic defined raffinose medium for 16–24 h prior to isolating mitochondria. Mitochondrial proteins (100 μg) were separated by SDS-PAGE and transferred to nitrocellulose. Strips were cut from a single blot and probed with the indicated antibodies against mitochondrial proteins (Aco1, Nfs1, Mir1, Yfh1, and Isu1). Cytochrome *c* (Cyc1) was analyzed using a duplicate gel. (C) Iron homeostasis in the frataxin-bypass suppressors. The indicated strains were grown in air for six doublings in defined raffinose medium in the presence of 10 μM ^55^Fe ascorbate. Cells were washed free of radioactive iron and separated into mitochondria and post-mitochondrial supernatant. An aliquot of mitochondria was lysed in the presence of 0.1% Triton X-100 for 10 min at room temperature, and supernatant (soluble) and pellet (insoluble) fractions were separated by centrifugation at 20,000 x *g* for 30 min at 4°C. Iron content was reported for whole cells (pmol per million cells), or various cellular fractions (pmol per micrograms protein).

The frataxin-bypassing activity of *ISU1* protein with changes of Met 141 to Cys, Ile, Val or Leu was shown by growth enhancement of the *YFH1* shuffle strain. In this strain, the chromosomal *ISU1* and *ISU2* were still intact (Fig 1B). To examine and compare these effects in more detail, matched strains were constructed in which each of the bypassing *ISU1* alleles was expressed in the absence *ISU2* (Table 1). The starting strain was designated *YFH1* [*ISU1*], indicating a *YFH1* positive strain with plasmid carrying wild-type *ISU1* (Met at position 141) (Table 1). Another strain was designated Δ*yfh1* [*ISU1*], indicating a strain deleted for *YFH1* and carrying wild-type *ISU1*. Similarly, Δ*yfh1* [*ISU1*-Cys], Δ*yfh1* [*ISU1*-Ile], Δ*yfh1* [*ISU1*-Leu], and Δ*yfh1* [*ISU1*-Val], were used to designate deletions of *YFH1* with the indicated *ISU1* mutant alleles (Table 1). The growth of the *YFH1* [*ISU1*] strain was more rapid than the Δ*yfh1* [*ISU1*] strain as shown by the larger colony size (Fig 2A, upper panel, rows 1 and 2). Hydrogen peroxide exposure exacerbated the Δ*yfh1* phenotype [37], further slowing growth and viability as a consequence of increased oxidative stress (Fig 2A, lower panel, rows 1 and 2). The *ISU1* alleles with Cys, Leu, or Val at position 141, similar to the *ISU1* M141I allele, improved growth of Δ*yfh1* under these conditions, including in the presence of hydrogen peroxide, although not quite to levels in the frataxin plus strain (Fig 2A, upper and lower panels, rows 1–6).

In terms of mitochondrial proteins, immunoblotting with anti-frataxin antibody confirmed the correctness of the genetic assignments: the *YFH1* strain expressed frataxin (Fig 2B, Y-M, lane 1) and the *Δyfh1* strains did not (Fig 2B, lanes 2–6). The *ISU1* alleles were all expressed in mitochondria. The abundance of Isu1 protein was increased in the Δ*yfh1* [*ISU1*] strain in the presence of the *ISU1*-Met allele (Fig 2B, Δ-M, lane 2), but in the presence of the bypassing *ISU1* alleles, Isu1 expression returned to normal, similar to the *YFH1* positive strain (Fig 2B, lanes 3–6). The changes in protein abundance were several fold, and most likely attributed to Aft1/2 transcriptional effects and protein stability effects [32]. Nfs1 protein levels were unchanged in all the strains consistent with the lack of regulation of the protein level (Fig 2B), although cysteine desulfurase activity was strongly regulated (see below).

Cytochrome *c*, a heme protein, was undetectable in the Δ*yfh1* [*ISU1*] strain (Fig 2B, lane 2) compared with the wild-type (Fig 2B, lane 1). The various suppressor strains recovered significant levels of cytochrome *c* (Fig 2B, lanes 3–6). Cytochrome *c* is regulated transcriptionally and post-transcriptionally by heme availability [38]. The dramatic changes in protein abundance probably reflect changes in heme synthesis and steady state heme levels.

Iron homeostasis was examined using steady state labeling of growing cells with radioactive ^55^Fe. In *YFH1* [*ISU1*] strain, cellular iron levels were appropriately regulated, whereas in the mutant Δ*yfh1* [*ISU1*] strain, by comparison, excess iron accumulated (Fig 2C, Cellular iron, Y-M versus Δ-M). The presence of the suppressor *ISU1* mutant alleles, *ISU1*-Cys, *ISU1*-Ile, *ISU1*-Leu, or *ISU1*-Val, restored cellular iron levels towards normal (Fig 2C, Cellular iron, Δ-C, Δ-I, Δ-L, Δ-V). The radiolabeled cells were subjected to subcellular fractionation, separating mitochondrial and post-mitochondrial fractions (Fig 2C, Mitochondrial iron and Post-mito supernatant iron). The iron quantitation for these fractions resembled the patterns for whole cell iron. The radiolabeled mitochondrial iron showed features that were dependent on *YFH1*. In the *YFH1* [*ISU1*] mitochondria, the predominant portion was solubilized by exposure to non-ionic detergents such as Triton X-100, whereas in the Δ*yfh1* [*ISU1*] mitochondria, proportionally more iron was recovered in the pellet following centrifugation in the presence of detergent (Fig 2C, Mitochondrial iron distribution, Y-M versus Δ-M). Most likely this effect reflects the accumulation of nanoparticles of ferric phosphate that is a hallmark feature of *Δyfh1* mitochondria [25]. In mitochondria from the different suppressor strains the amount of insoluble iron was decreased compared with the control deletion strain Δ*yfh1* [*ISU1*], indicating an improvement in the biochemical properties of mitochondrial iron pools (Fig 2C, Mitochondrial iron distribution, Δ-M versus Δ-C, Δ-I, Δ-L, Δ-V).

**Fig 3:**
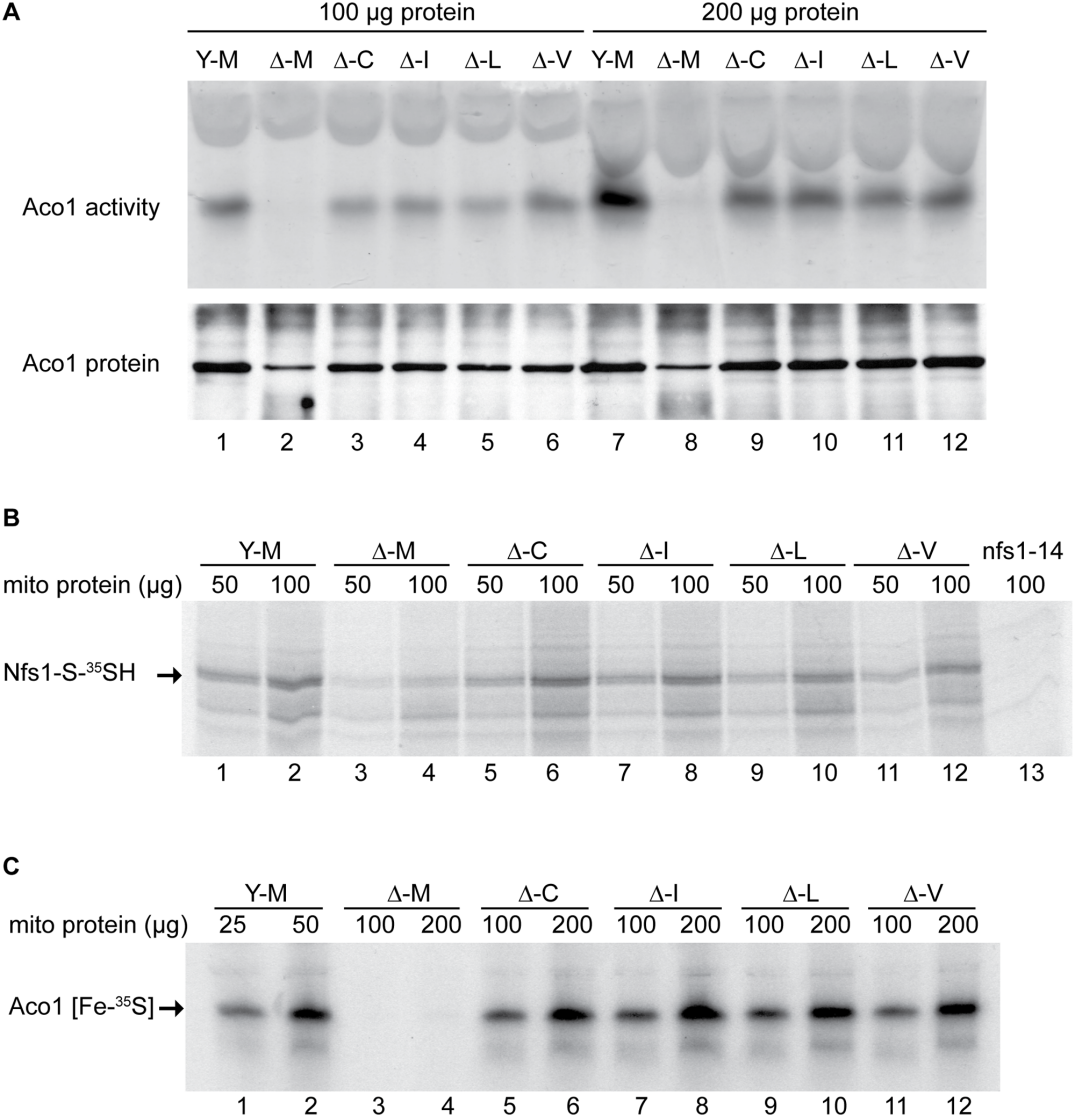
Assessment of Fe-S cluster assembly in mitochondria isolated from frataxin-bypass mutants. The same strains as in Fig 2 were assayed as follows: (A) Aconitase activity by in-gel assay. Mitochondria were lysed and 100 μg (lanes 1–6) or 200 μg (lanes 7–12) proteins were separated by native gel and developed using reagents that reflect aconitase activity (upper panel). The same lysates were separated by SDS-PAGE and analyzed by immunoblotting with anti-aconitase antibody to show the level of total aconitase protein (lower panel). (B) Persulfide formation on Nfs1 in mitochondria. Isolated and intact mitochondria were depleted of nucleotides and NADH by incubation at 30°C for 10 min. Either 50 μg or 100 μg protein equivalents were labeled for 15 min with ^35^S-cysteine. Samples were diluted with buffer, mitochondria were recovered by centrifugation, and total proteins were analyzed by non-reducing SDS-PAGE and autoradiography. The persulfide Nfs1-S-^35^SH is indicated by an arrow. Mitochondria from the *nfs1-14* mutant was included as a negative control (lane 13), because this hypomorphic Nfs1 mutant has negligible persulfide-forming activity [16]. (C) New Fe-S cluster synthesis on apoaconitase in isolated mitochondria. Isolated and intact mitochondria, were labeled with ^35^S-cysteine in the presence of added 4 mM ATP, 1 mM GTP, 5 mM NADH and 10 μM ferrous ascorbate for 30 min at 30°C. Mitochondria were recovered and membranes were ruptured. After centrifugation, the supernatant fractions containing soluble mitochondrial proteins (corresponding to 25 or 50 μg for *YFH1* [*ISU1*] and 100 or 200 μg for the other strains) were separated by native gel prior to autoradiography. The arrow indicates the aconitase protein containing newly made radiolabeled [Fe-^35^S] clusters.

Aconitase (Aco1), an abundant [Fe_4_S_4_] protein of mitochondria, was evaluated. An in- gel assay showed the aconitase enzyme activities in mitochondrial lysates. The control strain *YFH1* [*ISU1*] (Fig 3A, upper panel, lanes 1 and 7, Y-M) showed concentration dependent activity. The frataxin minus strain Δ*yfh1* [*ISU1*] (Fig 3A, upper panel, lanes 2 and 8, Δ-M) had no detectable activity regardless of the concentration of lysate in the assay. The various suppressors (Fig 3A, upper panel, Δ-C, Δ-I, Δ-L, Δ-V, lanes 3–6 and lanes 9–12) recovered significant activity, although not entirely to wild-type levels. Aconitase protein (Fig 3A, lower panel) as detected by immunoblotting was present in *YFH1* [*ISU1*] control (lanes 1 and 7), and was decreased in the frataxin-minus Δ*yfh1* [*ISU1*] (lanes 2 and 8) mitochondria, perhaps because the apoprotein was turned over more rapidly by mitochondrial proteases. Aconitase protein levels were restored in the suppressor strains (Fig 3A, lower panel, lanes 3–6 and lanes 9–12), consistent with recovery of enzyme activity.

The persulfide-forming activity in these mitochondria was tested as an indication of their cysteine desulfurase activity. Isolated and intact mitochondria were depleted of endogenous nucleotides and NADH by incubation at 30°C for 10 min, thereby blocking Fe-S cluster biogenesis without disrupting cysteine desulfurase activity [16]. After labeling with ^35^S-cysteine, mitochondrial proteins were separated by non-reducing SDS-PAGE, and the persulfide covalently bound to Nfs1 was visualized by autoradiography (Fig 3B, Nfs1-S-^35^SH). The signal was absent in *nfs1-14* mitochondria, which were unable to form significant Nfs1 persulfide because of a hypomorphic mutation in Nfs1 (Fig 3B, lane 13) [16]. The specificity of the Nfs1-persulfide signal was further confirmed by immunoprecipitation with anti-Nfs1 antibody [16]. Several other radiolabeled bands were detected in mitochondria (Fig 3B), some of which were present in the *nfs1-14* control and some of which were absent. Thus these background bands could be attributed to direct binding of ^35^S-cysteine, persulfide transfer from Nfs1 which was incompletely blocked, or binding of other reactive cysteine persulfides to mitochondrial proteins. The *YFH1* [*ISU1*] mitochondria (Fig 3B, lanes 1 and 2, Y-M) had significantly more Nfs1 persulfide than the frataxin-minus mutant Δ*yfh1* [*ISU1*] (Fig 3B, lanes 3 and 4, Δ-M). Each of the suppressor mutants (Fig 3B, Δ-C, Δ-I, Δ-L, and Δ-V; lanes 5–12) recovered persulfide-forming activity in mitochondria. Differences among the suppressor alleles were not apparent, and each one provided rescue of the persulfide-forming activity that was deficient in Δ*yfh1* [*ISU1*] mitochondria.

Next, ^35^S-cysteine labeling of isolated intact mitochondria was performed in the presence of added ATP, GTP, NADH and iron, thereby permitting multiple cycles of Fe-S cluster formation to occur [16]. Soluble proteins from these mitochondria were separated on native gels, and autoradiography was used to detect newly synthesized Fe-S clusters on aconitase (Fig 3C, Aco1 [Fe-^35^S]). In the”wild-type” *YFH1* [*ISU1*] mitochondria (Fig 3C, lanes 1 and 2, Y-M) a strong signal was observed, whereas in the frataxin-minus Δ*yfh1* [*ISU1*] (Fig 3C, lanes 3 and 4, Δ-M) no signal was present, consistent with the important role of frataxin in mitochondrial Fe-S cluster assembly. In the various suppressor mutants, although they still lacked frataxin, Fe-S cluster synthesis on Aco1 was restored to 24–31% of the *YFH1* control as assessed by densitometry and correction for the total amount of protein loaded (Fig 3C, lanes 5–12).

### Random mutagenesis of *ISU1* and screening for additional frataxin-bypass suppressor alleles

**Fig 4:**
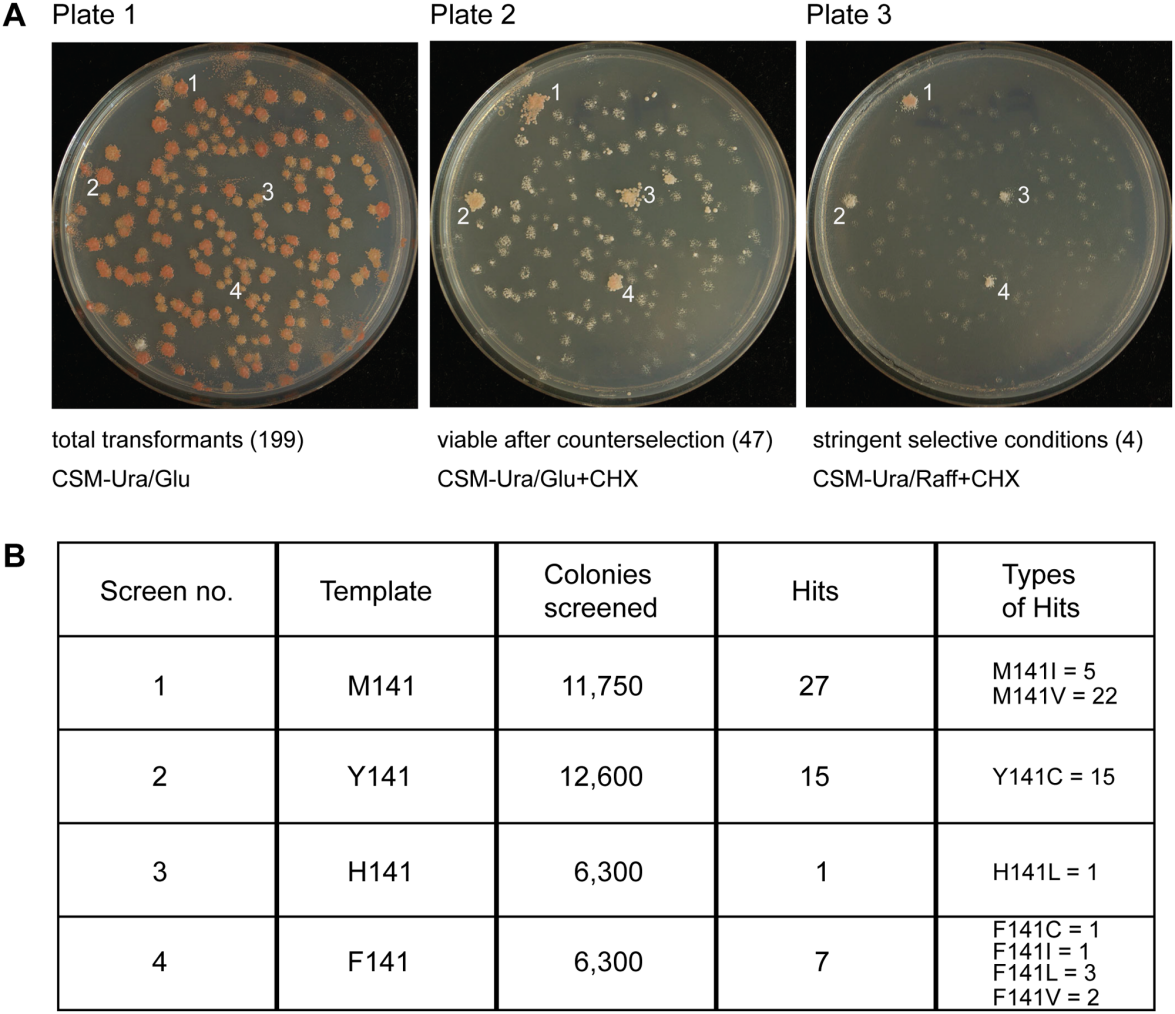
Screening of randomized *ISU1* in search of new frataxin-bypass suppressors reveals only amino acid substitutions of residue 141. (A) A library of linear *ISU1* sequences with random mutations was created by error prone PCR and co-transformed with a gapped plasmid (pRS416-*ISU1*) into a *YFH1* (Δ*isu1*) shuffle strain. The transformants contained recombined *ISU1* sequences with random mutations, and appeared uniform on defined uracil drop-out medium (plate 1). The transformants were replicated to cycloheximide and glucose containing plates, removing the covering pRS318-*YFH1* plasmid (plate 2). The transformants were also replicated to cycloheximide and raffinose containing plates (plate 3), selecting against the *Δyfh1* phenotype. (B) Summary table of screening results indicating starting template, number of colonies screened, number of hits conferring frataxin-bypass, and types of hits.

A library of randomly mutated *ISU1* plasmids was generated by error-prone PCR and transformed into a *YFH1* shuffle strain also deleted for *ISU1*. The colonies appeared uniform (Fig 4A, plate 1). The diversity of this library was confirmed by sequencing the inserts from randomly selected colonies. Of 42 colonies evaluated in this way, inserts were identified that included 72 amino acid changes in Isu1, which were well distributed throughout the coding region (S1 Table, controls). Transformants were replicated to cycloheximide plates, counterselecting against the *YFH1*-containing plasmid and uncovering the Δ*yfh1* phenotype. Most colonies grew slowly on glucose or not at all on raffinose, but a few colonies exhibited robust growth (Fig 4A, plates 2 and 3). Plasmid DNA rescued from these more rapidly growing Δ*yfh1* colonies was sequenced and in most cases was found to contain single nucleotide changes in *ISU1* conferring substitutions of residue 141 of the coding sequence. In some cases, *YFH1* sequences were found to have recombined into the *ISU1* plasmid and these clones were discarded. The PCR randomization was then repeated with different *ISU1* templates starting with Y141, H141 or F141, and a large number of colonies (approximately 36,950 representing 17,588 amino acid changes in Isu1) was screened in this way. The “hits” with frataxin-bypass activity included amino acid changes of M141 to Ile, Cys, Leu, and Val, sometimes in combination with other amino acids changes and sometimes alone (Fig 4B and S1 Table). However, no other amino acid change or combination of changes was able to confer frataxin-bypass activity. All possible amino acid changes in *ISU1* were not sampled, and only single nucleotide changes at position 141 were selected. The failure to find amino acid substitutions with bypass activity at other locations in the Isu1 protein may derive from the many constraints on this essential scaffold protein, which must interact with multiple partner proteins and perform multiple functions, such as stimulating Nfs1 activity, coordinating Fe-S clusters and transferring Fe-S clusters [39]. Based on these results, the possibility of finding another *ISU1* mutant with frataxin-bypass activity, while not entirely ruled out, seems unlikely.

### *E*. *coli* cross-species complementation and frataxin-bypass

The Met to Ile substitution in yeast *ISU1* that conferred frataxin-bypass activity did so by altering the methionine at position 141 (107 in the signal-cleaved mature protein) to the amino acid isoleucine used in the *E*. *coli* IscU in the corresponding position (I108 in IscU). Interestingly, deletion of the homologous frataxin gene *cyaY* in *E*. *coli* gave milder phenotypes in that organism than in yeast. Slowed growth, iron accumulation, and oxidative stress sensitivity were not observed in the *E*. *coli* knockout [27] in contrast to the yeast knockout. A series of species cross-complementation studies was undertaken in order to test the relative frataxin dependence or independence of yeast expressing the entire *E*. *coli* IscU protein targeted to mitochondria. For comparison, IscU protein was reverse engineered to place Met at position 108, as in the eukaryotic Isu1. The IscU protein (authentic *E*. *coli* protein with Ile) and IscU-Met (substituted *E*. *coli* protein with Met) separately were fused to the leader sequence of yeast mitochondrial protein CoxIV. Each of these fusion constructs was transformed into the *GAL1-ISU1/Δisu2* strain.

**Fig 5:**
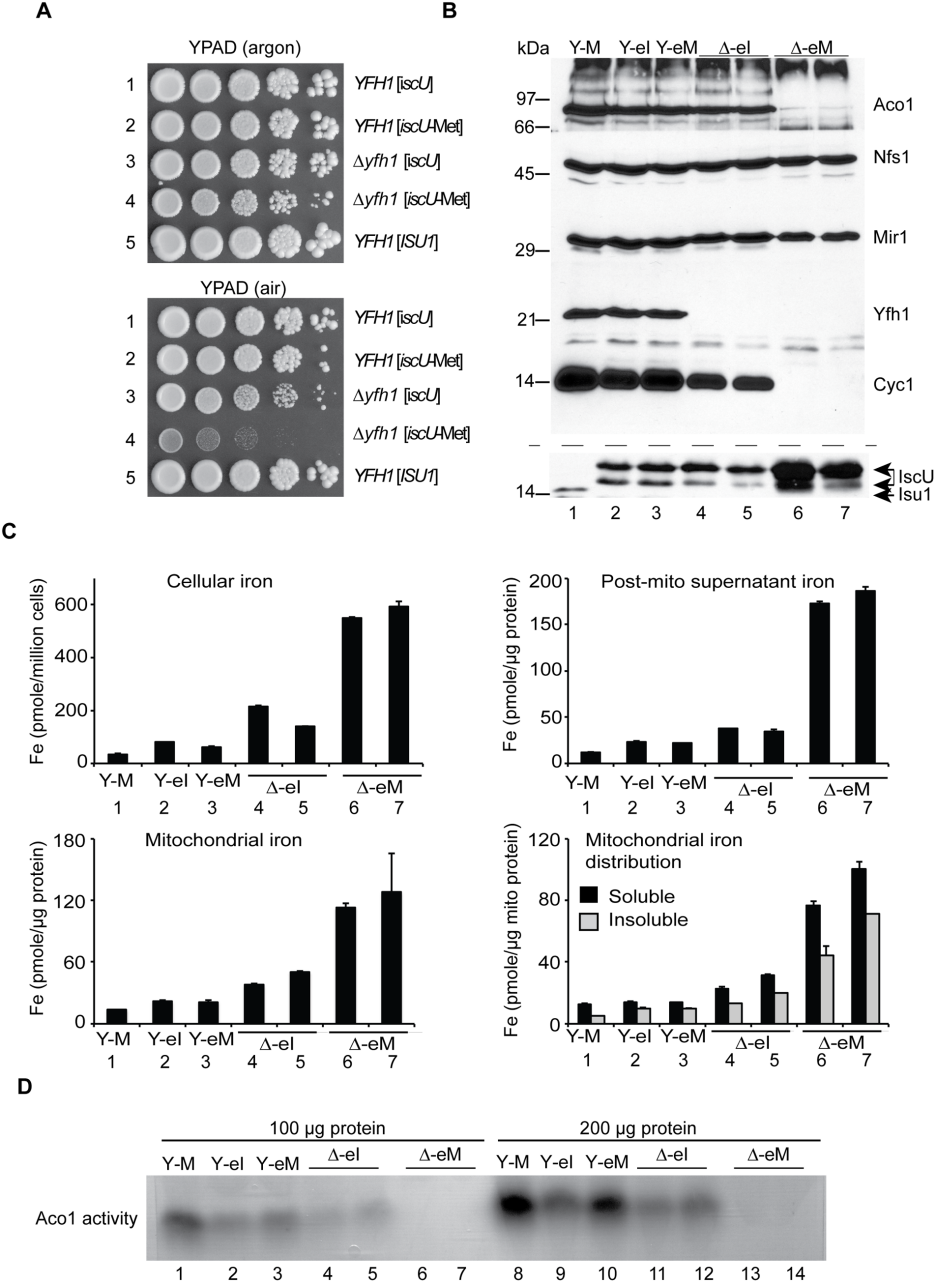
*E*. *coli iscU* or *iscU*-Met targeted to yeast mitochondria. (A) Growth phenotypes in argon or in air. Strains with mitochondrial targeted *E*. *coli* IscU proteins indicated in the figure were grown on YPAD in argon or in air for 3 days and photographed. Strain *YFH1 [ISU1]* was included as a control. (B) Mitochondrial protein levels by immunoblotting. The following strains were evaluated: 1) *YFH1* [*ISU1*] or Y-M, 2) *YFH1* [*iscU*] or Y-eI, 3) *YFH1* [*iscU*-Met] or Y-eM, 4) Δ*yfh1* [*iscU*] or Δ-eI clone 1, 5) Δ*yfh1* [*iscU*] or Δ-eI clone 2, 6) Δ*yfh1* [*iscU*-Met] clone 1 or Δ-eM, and 7) Δ*yfh1* [*iscU*-Met] clone 2 or Δ-eM. Cultures were inoculated into defined raffinose medium bubbled with argon. After an initial growth period the cultures were shifted to air. The doubling time of the Δ*yfh1* [*iscU*-Met] clones in air started at 2.5 h but prolonged to greater than 8 h, necessitating a longer growth period prior to harvesting these cells. Mitochondrial proteins were separated by SDS-PAGE, and transferred to nitrocellulose, which was cut horizontally into segments and probed with various antibodies against various mitochondrial proteins (Aco1, Nfs1, Mir1, Yfh1, and Cyc1). The segment used for anti-Cyc1 was stripped and reprobed with anti-Isu1. The antibody to the yeast Isu1 was used to detect the *E*. *coli* IscU. The two bands attributed to *E*. *coli* IscU are indicated by a double arrow, and yeast Isu1 is indicated by a single arrow. (C) Iron homeostasis. The indicated strains were precultured in argon-bubbled raffinose medium and then shifted to air in the presence of 10 μM ^55^Fe ascorbate. Following growth to a density of 2 x 10^7^ cells/ml, cells were harvested. Total cellular iron and iron in cellular fractions (post-mitochondrial supernatant, mitochondria, and soluble or insoluble fractions of mitochondria) were measured as in Fig 2C. (D) Aconitase activity. Mitochondria from the indicated strains were lysed, and 100 μg (lanes 1–7) or 200 μg (lanes 8–14) of proteins were separated by native gel and analyzed for aconitase activity using the in-gel assay.

The *E*. *coli* IscU proteins, with the authentic isoleucine or with the substituted methionine, were able to function in yeast as indicated by complementation of *GAL1-ISU1/Δisu2* cells. Each of these complemented strains grew well, indicating that the *E*. *coli* IscU or IscU-Met targeted to yeast mitochondria could function as the only Fe-S cluster assembly scaffold in the cell. No difference between *iscU* or *iscU*-Met expressing cells was noted in terms of growth (Fig 5A, rows 1 and 2). Frataxin was deleted in these strains, and a striking growth phenotype was observed. Both the Δ*yfh1* [*iscU*] or Δ*yfh1* [*iscU*-Met] could be maintained under an argon atmosphere with subtle differences in colony size on agar plates (Fig 5A upper panel). However, following air exposure, Δ*yfh1* [*iscU*] continued to grow with a doubling time of 2.5 h, whereas Δ*yfh1* [*iscU*-Met] progressively slowed until the doubling time reached 8.5 h in defined raffinose-based medium (Fig 5A, compare top panel for argon growth to bottom panel for aerobic growth in rows 3 and 4). This air/oxygen dependent growth inhibition was much more severe for the Δ*yfh1* [*iscU*-Met] strain than for the matched Δ*yfh1* [*ISU1*], carrying *Δyfh1* and yeast *ISU1*-Met. Perhaps the hybrid yeast-*E*. *coli* Fe-S cluster assembly machinery is particularly oxygen sensitive in the absence of frataxin. One of the functions of frataxin could be to shield the Fe-S cluster assembly machinery from oxygen [10].

Isolated mitochondria were examined for protein expression by immunoblotting. The yeast Isu1 migrated as a single band of about 14 kDa in mitochondria (Fig 5B, lane 1). The CoxIV fusions with the *E*. *coli* proteins reacted strongly with antibody raised against the yeast Isu1, giving rise to a slower migrating doublet (Fig 5B, lanes 2–7). The slower migrating band co-migrated with the bacterial expressed and purified IscU at about 17 kDa, and therefore it likely represents the CoxIV signal sequence-cleaved form of the IscU fusion protein. The more rapidly migrating form may be a proteolytic product. The retarded gel mobility of *E*. *coli* IscU compared with yeast Isu1 may be explained by its lower pI (4.7 for the *E*. *coli* IscU versus 9.3 for the yeast Isu1), which is associated with decreased binding of SDS and slower migration in the gel [40]. We have observed a similarly aberrant slow migration of Yfh1 due to its many acidic residues and failure to bind SDS [41].

In the Δ*yfh1* [*iscU*-Met] mitochondria, IscU protein was markedly increased in abundance (Fig 5B, lanes 6 and 7). By contrast, in the Δ*yfh1* [*IscU*] mitochondria, the IscU protein level was comparable to that of the frataxin plus strains (Fig 5B, lanes 4 and 5, compare with lanes 2 and 3). The difference may be traced to defective Fe-S cluster assembly in the Δ*yfh1* [*iscU*-Met] cells. In cells with impaired Fe-S cluster assembly, Isu1 protein abundance was previously shown to be up-regulated due to increased iron-dependent transcription mediated by the Aft1/2 regulator, and decreased turnover mediated by the Pim1 protease [32]. Furthermore, the *E*. *coli* IscU proteins might be poorly recognized by the yeast Pim1, further slowing turnover and increasing abundance (Fig 5B, lane 1 versus lanes 2 and 3). Other mitochondrial proteins were also examined. Nfs1 protein levels were comparable in all cases, consistent with the lack of regulatory changes (Fig 5B). Yfh1 was detected in the cells consistent with the predicted genotypes, being present in *YFH1* [*ISU1*], *YFH1* [*iscU*], and *YFH1* [*iscU*-Met] but absent in Δ*yfh1* [*iscU*] and Δ*yfh1* [*iscU*-Met] (Fig 5B). Cytochrome *c*, an indicator of cellular heme status, was present in the Δ*yfh1* [*iscU*] mitochondria expressing the authentic *E*. *coli* protein but completely undetectable in Δ*yfh1* [*iscU*-Met] mitochondria expressing the substituted form of the *E*. *coli* protein (Fig 5B, compare lanes 4 and 5 versus lanes 6 and 7).

Iron homeostasis was also evaluated. Two independent clones of Δ*yfh1* [*iscU*-Met] cells were tested because of the genetic instability and changeable phenotypes associated with this genotype. Both clones exhibited strongly increased cellular iron uptake compared with the unsubstituted control clones Δ*yfh1* [*iscU*] (Fig 5C, Cellular iron, bars 6 and 7). Iron accumulated in the post-mitochondrial supernatant and mitochondria of the Δ*yfh1* [*iscU*-Met] strains (Fig 5C, Post-mito supernatant and Mitochondrial iron, bars 6 and 7). The increase in mitochondrial iron levels was about 2–4 fold more than in the matched Δ*yfh1* [*iscU*] strains (Fig 5C, Mitochondrial iron, bars 4 and 5). Iron accumulated in both soluble and insoluble forms as assessed by centrifugation in the presence of Triton X-100, probably indicating ferric phosphate nanoparticle accumulation [25,26]. In all cases, the Δ*yfh1* [*iscU*-Met] mitochondria accumulated more insoluble iron than the Δ*yfh1* [*iscU*] mitochondria [Fig 5C, Mitochondrial iron distribution, bars 6 and 7 versus bars 4 and 5). In the frataxin-plus strains expressing *E*. *coli* proteins, *YFH1* [*iscU*] and *YFH1* [*iscU*-Met], iron homeostasis was mostly preserved (Fig 5C, all panels, bars 2 and 3). The most severe loss of iron homeostasis occurred in the Δ*yfh1* [*iscU*-Met] strain and correlated with the concurrent severe deficiency of Fe-S cluster proteins. The mechanism by which defective mitochondrial Fe-S cluster assembly perturbs iron homeostasis is still poorly defined. However, it is likely that important roles are played by loss-of-function of Fe-S cluster binding proteins such as Aft1/2 [42] and glutathione reductases [6].

Aconitase activity, measured by the in-gel assay of mitochondria, was present in Δ*yfh1* [*iscU*] mitochondria from two independent clones (Fig 5D, lanes 4, 5 and lanes 11, 12) and absent in Δ*yfh1* [*iscU*-Met] mitochondria from two independent clones (Fig 5D, lanes 6, 7 and lanes 13, 14). Aco1 protein was present in normal amounts in Δ*yfh1* [*iscU*] but markedly decreased in Δ*yfh1* [*iscU*-Met] mitochondria, consistent with increased turnover of the apoprotein (Fig 5B). By contrast, in frataxin plus mitochondria, aconitase protein and activity were present in all cases. Aconitase activity was greatest in the frataxin plus *ISU1* expressing yeast mitochondria (Fig 5D, lanes 1 and 8), slightly less in the frataxin plus *E*. *coli iscU* expressing mitochondria (Fig 5D, lanes 2, 3 and lanes 9, 10), and slightly less again in the frataxin null *iscU* expressing mitochondria (Fig 5D, lanes 4, 5 and lanes 11, 12). Thus aconitase activity correlated well with other features of these strains, including growth, cytochrome *c* levels, and iron homeostasis.

### Bioinformatic survey of Isu1/IscU

Isu1/IscU entries in the public database RefSeq (6064 in total) were collected and aligned on the highly conserved 12 amino acid sequence LPPVK LH CS**X** LA, using the Muscle algorithm [43]. The sequences were then sorted according to the amino acid at position X, where X is Met in the yeast Isu1 and Ile in the Isu1 suppressor mutant (S2 Table).

**Fig 6:**
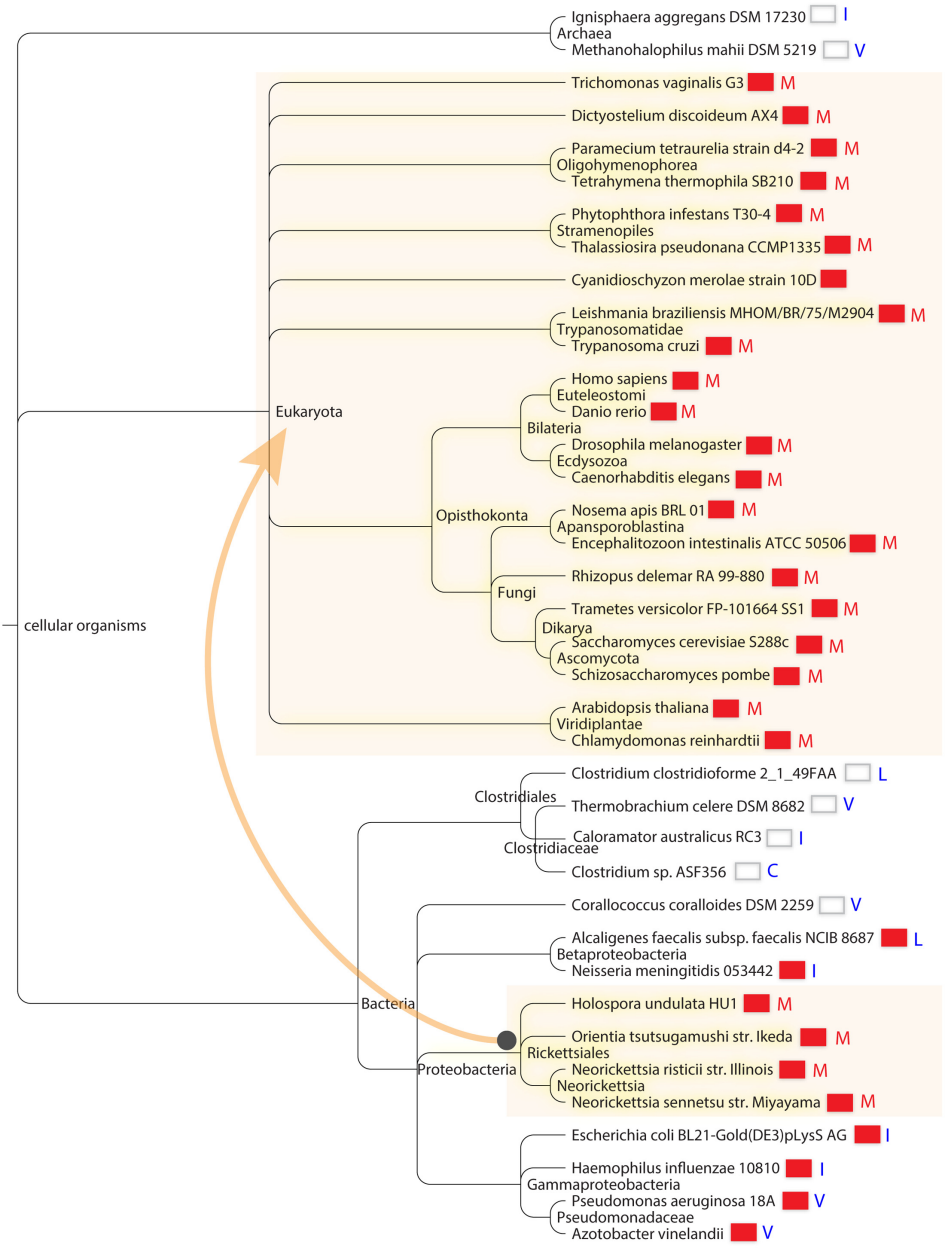
Cosegregation of Isu1/IscU-M and frataxin in global taxonomy. Distribution of species containing M (red letter) versus C I L V (blue letters) at the **X** position of the motif LPPVK LH CS**X** LA and correlation with the presence of frataxin. The presence of frataxin is indicated by a red colored box next to each species in the taxonomic tree. The depicted species were chosen to maximize diversity. The species are displayed using the NCBI Taxonomy browser according to their positions in the NCBI Taxonomy Database. The arrow connecting shaded areas in Proteobacteria and Eukaryota represents the direction of possible horizontal gene transfer occurring during mitochondrial creation.

Sorting on this position revealed highly interesting groupings. Firstly, we found that Isu1/IscU sequences with Met were present almost exclusively in eukaryotic species (302 of 307 entries, S2 Table). The converse was also true i.e. eukaryotic species had Isu1/IscU with Met present in almost all cases. The proteins with Met at position X were found in the most diverse branches of eukaryotes, including Excavata that lack classical mitochondria, Chromalveolata, various yeasts including Zygomycota, Basidiomycota, Ascomycota, land plants, photosynthetic single-celled organisms, various metazoans including worms, fish, flies, mice and humans (Fig 6). The only significant exceptions to the rule that Isu1/IscU with Met occurs in eukaryotes were several proteobacterial rickettsial species, including *Holospora undulata*, *Neorickettsia risticii*, *Neorickettsia sennetsu*, and *Orientia tsutsugamushi*. These organisms are intracellular parasites that are the closest living relatives of mitochondria. Interestingly, all these species have retained frataxin in their genomes [1], suggesting that IscU-Met and frataxin may have been co-inherited with the rest of the Fe-S cluster assembly machinery during the endosymbiotic event that gave rise to mitochondria (Fig 6) [1].

The lists of Isu1/IscU proteins using Cys, Ile, Leu, and Val at position X, i.e. those amino acid substitutions of yeast Isu1 conferring frataxin-bypass activity, included predominantly prokaryotic organisms. The Cys list had only 6 entries, all from bacteria, including several *Clostridium* species and an uncultured archeon (S2 Table). For the Ile list, almost all of the species were from prokaryotes (171 of 178 entries). The exceptions, i.e. eukaryotic species on this list, were interesting in that they often possessed more than one Fe-S cluster assembly scaffold gene. For example, the eukaryotic bumble bee, cucumber, armadillo and a single type of Mediterranean fly each carried Isu1 proteins with Ile at position 141, but the same organisms possessed another gene which used Met, as is typical for almost all eukaryotes. The Val column had almost exclusively prokaryotic or archaeal species (341 of 348 entries). In this column, the only eukaryotic species included *Entamoeba histolytica* and *Giardia intestinalis*, organisms that are known to have acquired Fe-S cluster assembly components from bacteria by gene transfer [44]. A single eukaryotic plant species, wheat or *Aegilops tauschii*, was found in this column. However, wheat also had another Isu gene using Met, and so it follows the theme of retaining more than one type of Isu, perhaps for adaptive advantages. Along the same lines, *Bos mutus*, a yak with a high altitude habitat, carried an Isu with Ile but also retained the more typical eukaryotic Isu with Met. The IscU proteins with leucine were found predominantly in prokaryotic species (181 of 199 entries). The outlier eukaryotic species on this list included selected apicomplexa (e.g. *Plasmodium falciparum*, *P*. *vivax*, *P*. *yoelli*), fungi (e.g. *Cryptococcus* and *Aspergillus* species) and mitosome containing microsporidia (e.g. *Nematocida parisii*).

The diversity of Isu1/IscU proteins is further increased by the expression of different splice forms in some species. Alternatively spliced forms of the scaffold protein genes were found in humans and armadillo. In humans, the X1 splice form inserts an Arg, and isoform X4 inserts a Lys at position X. The Lys containing isoform has been associated with destabilized protein and development of a human disease characterized by exercise intolerance and mitochondrial myopathy [45]. The substitutions of the Met 141 in yeast Isu1 with Lys and Arg were tested, and neither one was able to support scaffold activity or frataxin-bypass activity (Fig 1B). Nonetheless, it is still possible that these splice forms with Lys or Arg amino acid substitutions could serve a special function or afford an adaptive advantage under some special circumstances or in specific tissues.

In summary, Isu1/IscU amino acid sequences with Met at position X of the motif LPPVK LH CS**X** LA were predominantly found in eukaryotes but also occurred in several *Rickettsia* species. All contained frataxin in their genomes. Isu1/IscU proteins with the amino acids Cys, Ile, Val or Leu at position X were present primarily in prokaryotes, some with and some without frataxin in their genomes (Fig 6).

## Discussion

Frataxin was initially identified by reverse genetics after the Friedreich’s ataxia disease gene was cloned in 1996 [2]. Soon afterwards the protein was linked to iron metabolism by characterization of the striking iron homeostatic phenotype of the yeast deletion strain [23]. However, a more detailed understanding of frataxin’s function has been elusive. Recently, frataxin has been convincingly implicated in mitochondrial Fe-S cluster assembly [10]. Frataxin interacts with components of the mitochondrial Fe-S cluster machinery, including Nfs1, Isd11 and Isu1 [5]. It is vital for formation of Fe-S cluster intermediates on the Isu1 scaffold, a key step in the Fe-S cluster assembly process [3]. A suppressor Isu1 carrying the amino acid substitution Met to Ile at position 141 can correct or bypass most of the *Δyfh1* phenotypes that result from lack of frataxin [28]. The change introduces the amino acid used by *E*. *coli* in the highly homologous IscU protein. The *Δyfh1* yeast deletion strain is severely compromised and slow growing, whereas the *E*. *coli* strain deleted for the homologous frataxin protein is mildly compromised and grows normally. Here we have delved into the phenomenon of bypass of frataxin deletion, performing a number of genetic and biochemical experiments.

### The suppressor Isu1 acts as a genetic dominant and requires Nfs1 for its activity

The suppressor *ISU1*-Ile bypassed the requirement for *YFH1* when it was introduced on a plasmid. Similarly, the corresponding substitution in a plasmid carrying the paralogous *ISU2*-Ile conferred bypass activity. The effects were observed with chromosomal *ISU1* and *ISU2* genes remaining intact, thereby reflecting genetic dominance and suggesting a gain of function. It is therefore interesting to consider more specifically the nature of the gained function. One explanation could be that Nfs1/Isd11 exhibits only basal cysteine desulfurase activity, and that frataxin is needed to act as a positive effector, thereby inducing the optimal activated level of cysteine desulfurase [13,29]. The wild-type Isu1 has no stimulatory activity on its own and may even be inhibitory [13,33], but the suppressor Isu1-Ile is able to substitute for frataxin by stimulating the Nfs1/Isd11 cysteine desulfurase [29,30]. If both wild-type Isu1 and suppressor Isu1-Ile are present in the cell simultaneously, the stimulatory effect of the suppressor is the dominant effect, providing bypass activity. An implied consequence of this scenario is that the suppressor Isu1-Ile will require an active form of Nfs1 for it to be effective. Thus it makes sense that the hypomorphic allele of *NFS1*, *nfs1-14* [46], with decreased cysteine desulfurase activity, would not support bypass. The results provide genetic support for the hypothesis that frataxin and the bypass Isu1 work to produce their effects on Fe-S cluster assembly, at least in part, by boosting the activity of the cysteine desulfurase.

The suppressor Isu1 was shown to stimulate persulfide formation on Nfs1, similar to frataxin [29,30]. Subsequently, sulfur for Fe-S cluster synthesis must be transferred to the Isu1 scaffold and assembled with iron to form the Fe-S cluster intermediate. It is generally assumed that this process occurs in a protein complex with other Isu1 molecules present [35,36]. Therefore, we wondered if the Nfs1 stimulatory effect of the suppressor Isu1 could promote Fe-S cluster formation *in trans* on a normal copy of Isu1 or Isu2. However, at least in a series of genetic experiments, this was not the case. The survey of all the possible M141 amino acid substitutions identified a set of best scaffolds, moderate scaffolds and poor scaffolds, based on complementing activity in the *GAL1-ISU1/Δisu2* strain (Fig 1B). The same set of plasmids, scored for frataxin-bypassing capability, identified activity for M141 with Cys, Ile, Leu or Val changes, and these all fell into the top category for scaffold activity. Furthermore, if a second site substitution abolishing scaffold activity (e.g. changing a critical Cys to Ala) was introduced into the M141I-Isu1 protein sequence, and the doubly substituted Isu1 was introduced into a *Δyfh1* shuffle strain with wild-type *ISU1* and *ISU2* present, bypass activity was abrogated (Fig 1C). Thus most likely the suppressor Isu1 does not productively interact with the wild-type Isu1 to mediate bypass, but instead it replaces the wild-type Isu1 in providing both bypass and scaffold functions.

### Prokaryotic versus eukaryotic cysteine desulfurases

Eukaryotic and prokaryotic Fe-S cluster machineries are highly conserved. Both yeast and *E*. *coli* utilize cysteine desulfurases, scaffold proteins and frataxin homologs. However major differences in the frataxin deletion phenotypes have been reported, with essentiality or severely deleterious phenotypes in the eukaryotic case [4], and normal growth and relatively mild phenotypes in *E*. *coli* [27]. Why the difference? One possibility is that *E*. *coli* possesses a redundant Fe-S cluster assembly system, the SUF system, which may compensate for lack of frataxin [47]. However this does not entirely account for the phenotypic differences, because the SUF system is not generally deployed under standard growth conditions. Significantly, the cysteine desulfurases show key differences. Most prokaryotic cysteine desulfurases such as IscS are constitutively active, although some regulatory changes in activity have been described [15]. The eukaryotic cysteine desulfurases, on the other hand, seem to be largely inactive in their basal state. Activation is required, and this activation involves frataxin. Purified Nfs1 is able to bind the substrate cysteine in its PLP containing substrate-binding site. However, binding is inefficient, and frataxin interaction increases exposure and utilization of substrate-binding sites of the enzyme [29]. The suppressor Isu1-Ile protein is able to generate a similar alteration of Nfs1, mimicking the effect of frataxin on Nfs1, and providing a plausible explanation for its frataxin-bypass activity. Nfs1 enzyme with the substrate bound must still undergo another activation step, mediated by Isd11. Isd11 triggers a conformational change and persulfide formation, thereby generating the intermediate for Fe-S cluster assembly [29]. Here we have seen that not only the Ile but also the Cys, Val, Leu substituted forms of Isu1 are able to stimulate persulfide formation on Nfs1 in the absence of frataxin. Thus these bypassing alleles of Isu1 activate Nfs1 independently of frataxin, rendering the mitochondria more prokaryote-like.

### Frataxin-bypassing amino acid substitutions in Isu1 and how they may work

More than 17,000 amino acid changes of *ISU1* were surveyed, but only a small subset of those conferred bypass activity. In all cases, the active changes altered the amino acid at position 141 of Isu1, introducing the amino acids Cys, Ile, Leu, or Val. The mutagenesis was not exhaustive, but nonetheless, the data support the very restricted nature of the changes that confer this activity. The biochemical features of these newly discovered bypass mutants (Isu1 with Cys, Leu, or Val substituted at position 141) were similar to the original one (Isu1 with Met replaced by Ile). The various mutant forms of Isu1 conferred improved growth in the absence of frataxin and more efficient Fe-S cluster assembly in mitochondria. The persulfide-forming activity was increased in mitochondria lacking frataxin, indicating enhanced cysteine desulfurase activity.

**Fig 7:**
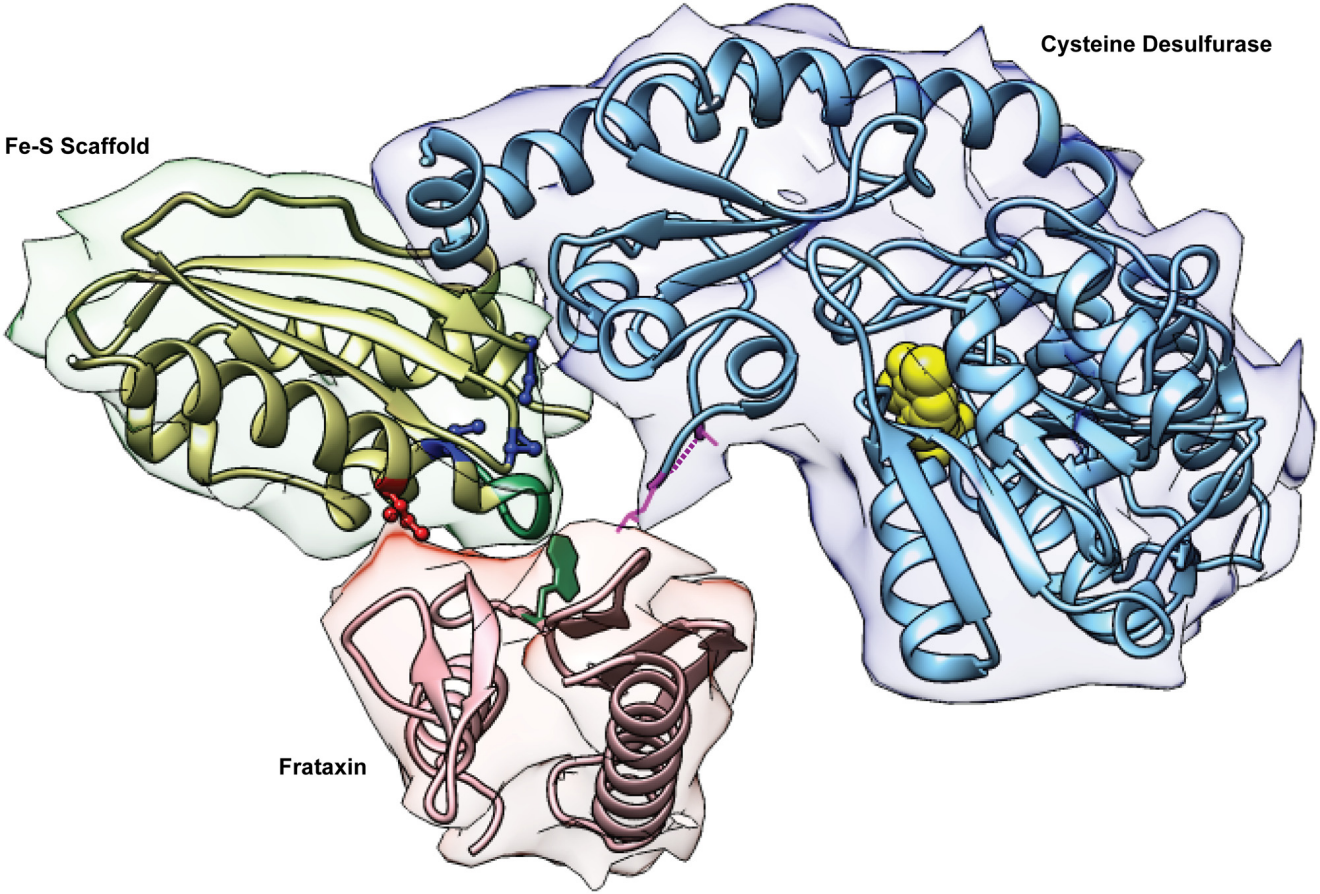
The Fe-S cluster assembly complex of *E*. *coli*. The Protein Data Bank (PDB) file of the bacterial Fe-S cluster assembly complex [36] was kindly provided by Annalisa Pastore (King’s College London). The image was drawn using the Stanford Chimera program. The cysteine desulfurase is IscS for *E*. *coli* or Nfs1 for yeast; frataxin is CyaY for *E*. *coli* or Yfh1 for yeast; the Fe-S scaffold is IscU for *E*. *coli* or Isu1/Isu2 for yeast. Highlighted features of IscU/Isu1 are the suppressor amino acid Ile (red), the Fe-S cluster cysteine ligands (blue), and the PVK frataxin-binding site (green). The reciprocal interaction site on frataxin includes a tryptophan (green). The PLP cofactor of IscS/Nfs1 is shown as yellow spheres, and a dotted purple line is used to indicate the approximate position of the active site mobile loop, which is not seen in the structures.

How does the Isu1 suppressor work? Isu1 is a central component of the Fe-S cluster assembly complex consisting of Nfs1/Isd11/Isu1/Yfh1. The components are highly conserved with their bacterial homologs, with the exception of Isd11. Structural information has been obtained only for the bacterial components [11,36]. For Isu1/IscU the structure includes alpha helices framing a platform of beta sheets, with three conserved cysteines oriented towards a binding pocket in the core and able to coordinate the Fe-S cluster intermediate (Fig 7). The amino acid motif LPPVK is found towards the beginning of a long C-terminal alpha helix [11,39]. Interestingly, the PVK motif (Fig 7, green in Fe-S scaffold) was shown to include the frataxin-binding site [33]. The suppressor residue Ile (Fig 7, red ball-and-stick) is predicted to lie on an exposed surface of this helix, opposite the Fe-S liganding Cys, which is found on the opposite interior face of the helix (Fig 7, blue ball-and-stick). Thus the Met to Ile amino acid change near to this frataxin-binding site on Isu1 might mimic the effects of frataxin binding. Substitutions of Cys, Val, or Leu would be predicted to produce similar changes.

Conformational changes of the Fe-S cluster assembly complex facilitating Fe-S cluster intermediate formation might ensue. Nfs1 might be altered in a way that exposes substrate-binding sites for interacting with cysteine [29]. (Fig 7, yellow balls for PLP in the binding site). The sulfur intermediate, converted to a persulfide and bound to the flexible loop of Nfs1 (Fig 7, magenta dotted line and adjacent residues), might be more readily transferred to the modified Isu1. The Cys 139, an Fe-S cluster ligand on Isu1, that is one helix turn away from the Ile suppressor residue, might be rendered more accessible for persulfide transfer (Fig 7). Iron delivery remains the least characterized step in Fe-S cluster formation. Frataxin might facilitate iron entry into the Fe-S cluster assembly complex by initiating a conformational change that promotes transfer of mitochondrial iron from a physiological ligand (still undefined) to Isu1. Alternatively, frataxin might play a more direct role in binding iron and delivering it to Isu1 as has been shown for Yfh1 [19]. The Isu1-Ile protein (or bacterial IscU) might accomplish a similar function by exposing iron-binding sites on Isu1 [19]. The iron delivery step in Fe-S cluster assembly will need to be better understood to support or to refute these possibilities.

### Iron homeostasis and heme

Iron homeostasis was improved, correlating with the improvements in cytochrome *c*, a mitochondrial heme protein. In previously published work, cytochromes were virtually absent in *Δyfh1* and restored by the *ISU1*-Ile allele as assessed by low temperature spectra of whole cells [28]. Other heme proteins such as cytochrome *c* peroxidase, Ccp1, were similarly affected [30]. Heme synthesis, measured by ^55^Fe incorporation into porphyrin in isolated mitochondria, was very low in Δ*yfh1* and recovered in the presence of the substituted *ISU1* [28]. What is the mechanistic connection between a change in the Fe-S cluster assembly scaffold and these effects on heme synthesis? Heme synthesis occurs inside mitochondria and involves the insertion of iron into porphyrin by the enzyme ferrochelatase to make protoheme. Porphyrin and ferrochelatase activity are not lacking, and in fact, zinc protoporphyrin accumulates in the *Δyfh1* mutant [25]. Instead iron is the likely source of the trouble. Iron accumulating in Δ*yfh1* mitochondria (and in other Fe-S cluster deficient cells) exhibits changes in its physical properties and solubility that could lead to loss of bioavailability [25]. The data suggest that Fe-S cluster synthesis acts upstream of heme synthesis in promoting iron bioavailability for heme synthesis, although many mechanistic details are still lacking.

### Taxonomy of Isu1/IscU sequences with or without frataxin

Isu1/IscU amino acid sequences sorted according to the amino acid appearing in position 141 fall into clearly defined grouping, underscoring the importance of this residue for Isu1/IscU function. The amino acid Met appeared almost exclusively in eukaryotic organisms. The only significant exceptions were several prokaryotic *Rickettsia* species, which were classified as proteobacteria. In all of these organisms with IscU-Met, frataxin was also present in their genomes. We can imagine a scenario in which IscU-Met and frataxin originated together in proteobacterial ancestors of mitochondria such as *Rickettsia*, and from there gave rise to modern mitochondria via horizontal gene transfer during the endosymbiotic event (Fig 6). A key point is that the Met amino acid was found only in organisms with frataxin, as if Met serves to “lock in” frataxin by ensuring frataxin dependence of Fe-S cluster assembly. The co-dependence of these two elements, the IscU-Met and frataxin, appears to be quite profound, as they have been retained together throughout all the eukaryotic branches of the tree of life. In the prokaryotic world, on the other hand, various IscU variants were found, and various amino acids were found at position X adjacent to the PVK frataxin-binding motif of the IscU homologs. The amino acids Cys, Ile, Leu, or Val which conferred frataxin-bypass in biochemical experiments in yeast, were found in IscU proteins of species with or without frataxin homologs (Fig 6). Thus the evolutionary record suggests that these amino acids may be associated with relative frataxin independence of Fe-S cluster assembly. The advantages of retaining IscU-Met and frataxin versus IscU-Ile and no frataxin remain to be ascertained, as both arrangements are able to support efficient and regulated Fe-S cluster assembly.

### Implications for Friedreich's ataxia

Friedreich’s ataxia is a progressive degenerative disease affecting neurons such as dorsal root ganglia and cardiomyocytes, and certain other tissues. The disease is caused by frataxin deficiency in affected tissues and is associated with defective Fe-S cluster assembly [24,48]. The efficacy of frataxin-bypass in yeast can be viewed as a reprogramming of mitochondrial Fe-S cluster assembly to a more prokaryotic type, such that the cysteine desulfurase and other features of the assembly process become more frataxin-independent. The discovery of several substitutions, changes to Cys, Ile, Leu, or Val, that are able to confer frataxin-bypass suggests that there may be common structural features of these Isu proteins that could be mimicked by small molecules [49]. The genetic dominance of the suppressor activity in the *Δyfh1* yeast also suggests that such an approach could be effective in a therapeutic setting in human mitochondria where frataxin is deficient and normal Isu1 is still present.

## Materials and Methods

### Construction of Isu1 mutant plasmids and yeast strains

A set of plasmids was generated containing modified versions of the Isu1 coding sequence (between NdeI and XhoI), carried between the native Isu1 promoter (700 bp between EagI and NdeI) and terminator (200 bp between BamHI and SacI) on a centromere based plasmid, YCplac22. Residue 141 of the full length Isu1 precursor protein was changed from M to each of the other 19 amino acids, using QuikChange mutagenesis (Agilent Technologies Inc.). In addition, N123D and N123A mutants were generated. Cysteine mutants of the Isu1 coding sequence were constructed in which C69, C96 and C139 were each changed to A. The M141I change was then introduced into each of these cysteine mutants, creating plasmids C69A-M141I, C96A-M141I, and C139A-M141I. A cysteine swap mutant was created in which C139A was combined with M141C in the full length Isu1. The various mutant forms of Isu1 were tested for Yfh1-bypassing function and scaffold function by transforming into strains *YFH1* shuffle and *GAL1-ISU1/Δisu2*, respectively (Table 1). For testing genetic dominance of the *ISU1* bypass suppressor, the suppressor allele (YCplac22-*ISU1*-M141I) was introduced into strain 70–31 containing genomic copies of *ISU1* and *ISU2* (Table 1), and the covering *YFH1* plasmid was removed by fluoroorotic acid (FOA) treatment. For testing *ISU2* function, the native *ISU2* sequence was amplified from plasmid pGP564-*ISU2* including 700 bp 5’ and 200 bp 3’ of the coding sequence, and cloned between restriction sites HindIII and BamHI in YCplac22, making plasmid YCplac22-*ISU2*. The M133 was changed to Ile by site directed mutagenesis, creating YCplac22-*ISU2*-M133I. The *ISU2* coding sequence was inserted into NdeI-XhoI sites in place of the *ISU1* coding creating YCplac22-ISU2coding. The M133 was changed to Ile by site directed mutagenesis creating plasmid YCplac22-*ISU2*coding-M133I.

### Mutant strains expressing alleles of Isu1 (Cys, Ile, Leu, or Val) in single copy

Strain *GAL1-ISU1/Δisu2* was transformed with plasmid YCplac22-*ISU1* in which M141 was changed to C, I, L or V and the chromosomal *GAL1* promoter was turned off by shifting cells from galactose to glucose as the carbon source. The *YFH1* gene was deleted by transforming with plasmid pRS405-gamma-yfh1 linearized with BamHI, and selecting for transformants on defined leucine drop-out medium in an argon-filled chamber. These low oxygen conditions were previously shown to mitigate the mutant phenotype and allow for stable propagation of the knockouts [30]. The knockouts were confirmed by PCR of the *YFH1* locus. The strains were denoted Δ*yfh1* [*ISU1*] (115–26), Δ*yfh1* [*ISU1*-Cys] (116–54), Δ*yfh1* [*ISU1*-Ile] (115–28), Δ*yfh1* [*ISU1*-Leu] (116–53), and Δ*yfh1* [*ISU1*-Val] (116–51). Congenic wild-type strain *YFH1* [*ISU1*] was included as a control.

The strains were thawed from -80°C vials and inoculated to CSM-Trp/2% raffinose defined medium agar plates kept in an argon-filled jar. Cells were inoculated from the plates into liquid medium of the same composition. In general, small cultures of 50 ml were grown in argon-filled bottles for two days without shaking, expanded to 100 ml cultures while shaking in air, and then diluted again into 1 L cultures supplemented with 10 μM ferrous ascorbate. The 1 L cultures were grown at 30°C for 16 h, and the total time exposed to air was approximately 24 h for the slow-growing strains.

### Iron measurements and enzyme assays

Cellular and subcellular iron levels were determined by growing cells for at least four doublings in standard defined medium supplemented with 58 nM ^55^FeCl_3_, 10 μM unlabled ferric chloride and 100 μM ascorbic acid. Cells were washed free of unincorporated iron, and then they were ruptured by vortexing with glass beads in the presence of 50 mM Hepes/KOH, pH 7.5, 150 mM NaCl, 0.6 M sorbitol. Differential centrifugation was used to remove unbroken cells and to separate the remainder into mitochondrial and post-mitochondrial fractions [50]. The post-mitochondrial fraction included both cytoplasm and vacuoles, as no effort was made to separate these two cellular components. Mitochondria were shown to be mostly intact by evaluation of mitochondrial marker proteins, which remained with the mitochondrial fraction. Mitochondria were lysed for 10 min at room temperature in the presence of 0.1% Triton X-100 in hypotonic buffer (50 mM Hepes/KOH, pH 7.5, 150 mM NaCl). The supernatant (soluble) and pellet (insoluble) portions were separated by centrifugation at 20,000 x *g* for 30 min. Iron content was determined by scintillation counting for ^55^Fe, and protein content was measured by bicinchoninic acid assay (BCA, Pierce) [28]. For whole cells, iron content was reported as pmol iron per million cells, whereas for the cellular fractions iron content was reported as pmol iron per microgram protein. An in-gel activity assay for aconitase was performed. Briefly, mitochondria were lysed in buffer consisting of 50 mM Tris-HCl pH 8, 50 mM NaCl, 1% TX-100, 10% v/v glycerol, 2 mM Na-citrate and 15 U catalase. Samples were loaded on a native acrylamide gel containing 132 mM Tris base, 132 mM boric acid, 3.6 mM sodium citrate. The signal was developed in the gel by incubating in developing buffer containing 100 mM Tris-HCl, pH 8, 1 mM NADP, 2.5 mM cis-aconitic acid, 5 mM MgCl_2_, 1.5 mM methylthiazolyldiphenyl-tetrazolium bromide (MTT), 0.3 mM phenazine methosulfate, and 5 U/ml isocitrate dehydrogenase [51,52].

### Organelle assays for persulfide and Fe-S cluster synthesis

Persulfide formation on Nfs1 present in intact mitochondria was measured as described [16]. Isolated mitochondria were depleted for endogenous nucleotides and NADH by incubation for 10 min at 30°C in buffer (20 mM Hepes/KOH, pH 7.5, 0.6 M sorbitol). These mitochondria were incubated with ^35^S-cysteine (10 μCi) for 15 min at 30°C. Mitochondria were recovered, proteins were separated by non-reducing SDS gel, and the persulfide was viewed by radioautography. Fe-S cluster formation was measured as described [12]. Mitochondria were incubated with ^35^S-cysteine for 30 min in the presence of added ATP (4 mM), GTP (1 mM), NADH (5 mM) and iron (10 μM ferrous ascorbate). Mitochondria were recovered and the soluble proteins were released by freeze-thaw and sonication and separated on a native gel. The signal associated with newly formed [Fe-^35^S] aconitase was visualized by radioautography.

### Genetic screen for Isu1 alleles with bypass activity

Pools of mutagenized linear fragments of 1443 bp including the coding region of the *ISU1* gene were generated by mutagenic PCR in the presence of 1 mM MnCl_2_ and altered nucleotide concentrations (2 mM dATP, 2 mM dGTP, 10 mM dTTP, 10 mM dCTP). The primers used were: A (5’ TTTTTTCGGCCGTTCTTTTCTTTTTCTTGCACTACC 3’) and B (5’ TGATTTGAGCTCagcacgtccgtcccgctttcaccctgg 3’), and the templates were different YCplac22-*ISU1* plasmids with M, Y, H or F at position 141 of the coding region. Each mutagenized pool was co-transformed with a gapped plasmid (pRS416-*ISU1* digested with MscI and BamHI) into the *YFH1* shuffle strain 109–9 (*Δyfh1*::*TRP1 Δisu1*::*HIS3MX6 ISU2 cyh2 [pRS318-CYH2-LEU2-YFH1]*, Table 1). After selecting for transformants on uracil drop-out medium with glucose as the carbon source, the colonies were replicated to uracil drop-out, cycloheximide medium with glucose or raffinose as the carbon source. The large colonies appearing after several days on the raffinose plates were analyzed further. Colony PCR was used to check for absence of *YFH1*. Transformants still harboring *YFH1* after counterselection were discarded. The remaining clones were expanded, and the plasmid-borne *ISU1* alleles were rescued in *E*. *coli* and the DNA was sequenced.

### *E*. *coli* IscU constructs and yeast strains for mitochondrial targeting

The coding region of *E*. *coli* IscU was amplified from genomic *E*. *coli* DNA from strain DH5 alpha and inserted into the XbaI and XhoI sites of a YCplac22 derived plasmid. In this plasmid, the *ISU1* promoter consisting of 700 bp, is followed by the first 22 amino acids of the cytochrome *c* oxidase subunit IV (CoxIV) mitochondrial signal sequence [53], and 200 bp of *ISU1* terminator. The resulting plasmid was checked by DNA sequencing and confirmed to code for a CoxIV-IscU fusion protein with amino terminus as follows: MLSLRQSIRFFKPATRT^LCSSRH**MAYSEKVID**. The caret indicates the predicted signal sequence cleavage site by the mitochondrial processing peptidase, and the bolded letters indicate amino acids of *E*. *coli* IscU. The rest of the IscU sequence was confirmed to be the same as listed in Genbank accession No. AAJU02000016.1. The amino acid I108 of IscU (equivalent to M107 in signal-cleaved mature Isu1 or M141 in the full length precursor form of Isu1) was changed from I to M by QuikChange mutagenesis, generating a plasmid for expressing IscU-Met in mitochondria. The mitochondrial-targeted *iscU* plasmids were introduced into strain *GAL1-ISU1/Δisu2*, generating strains *YFH1* [*iscU*] and *YFH1* [*iscU*-Met]. *YFH1* was deleted in these strains by transforming with pRS405-gamma-yfh1, linearized at BamHI, followed by selection on leucine drop-out medium in an argon-filled anaerobic jar. This created strains *Δyfh1* [*iscU*] and Δ*yfh1* [*iscU*-Met] (Table 1). Deletion of *YFH1* was verified by PCR. Strain *GAL1-ISU1/Δisu2* transformed with YCplac22-*ISU1* served as the control strain *YFH1* [*ISU1*]. All strains were grown in an argon-filled chamber or in argon-bubbled medium, and cells were then exposed to air in defined raffinose medium for iron labeling and mitochondrial isolation.

### Bioinformatic analysis of Isu1/IscU protein sequences

Isu1/IscU related sequences were collected by using BLAST similarity to the Isu1 of *Saccharomyces cerevisiae* S288c from the RefSeq non-redundant database. All related entries in RefSeq (6064) were aligned using the Muscle program [43], according to the amino acid at position **X** (equivalent to Isu1 residue 141) of the amino acid motif LPPVK LH CS**X** LA. The lists were manually edited, and duplicates were removed, retaining one entry for each species. For each entry, the sequence was scored + if it contained 8 or more amino acids identical to the query, and 0 if contained 7 or less. The sequences were scored * for exceptions if they deviated from the rule that eukaryotic Isu proteins use only methionine at that amino acid position and prokaryotic Isu proteins do not use methionine at that position.

## Supporting Information

S1 Fig Frataxin-bypass by *ISU2*-Ile. The *Δyfh1* shuffle strain 70–31 strain was transformed with *ISU2*-containing plasmids and control plasmids, and the covering *YFH1* plasmid was removed by FOA treatment. The plasmids are: 1) YCplac22-*ISU2*coding-Met, 2) YCplac22-*ISU2*coding-Ile 3) YCplac22-*ISU2*-Met, 4) YCplac22-*ISU2*-Ile, 5) YCplac22, 6), YCplac22-*ISU1*-Met, 7) YCplac22-*ISU1*-Ile, 8) YCplac22-*YFH1*. Frataxin-bypass was observed for *ISU2*-Ile and *ISU2*coding-Ile in which the mutated coding sequence of *ISU2* was expressed from the *ISU1* promoter.
(TIF)

S1 Table List of randomly generated mutations of *ISU1* selected for frataxin-bypass activity. The *ISU1* coding sequence was amplified by error-prone PCR from the native sequence (M141) or from mutated sequences plasmids (Y141, H141 or F141) and co-transformed with a gapped *ISU1* plasmid into the *YFH1* shuffle strain. Following removal of the *YFH1* plasmid, clones with improved growth were identified, and the plasmid borne *ISU1* sequence was determined. The table lists amino acid changes in the *ISU1* coding of the “hits” with improved growth and the corresponding nucleotide sequence of the codon at position 141 of *ISU1*. Among the hits, M141I was selected 6 times, M141V was selected 24 times, M141L was selected 4 times, and M141C was selected 17 times. No selected clones had the wild-type sequence, M141. As a control, 42 unselected clones were evaluated from the randomized pool starting with M141, and these all retained the M141 codon.
(XLSX)

S2 Table Bioinformatic survey of Isu1/IscU sequences. Isu1/IscU proteins from the RefSeq non-redundant database (total number of 6064) were aligned according to the amino acid at position **X** of the 12 amino acid motif LPPVK LH CS**X** LA. In all, 21 lists were collected, one for each amino acid at position **X** and a no-alignment list. The lists were manually edited, retaining one entry for each species. For each entry, columns were annotated with the species name, accession number, taxonomic category, and the sequence of the 12 amino acid motif. The sequences were scored + if they contained 8 or more amino acids identical with the motif present in yeast Isu1 and 0 if they contained 7 or less identical amino acids. The sequences were scored * for exceptions if they deviated from the rule that Isu1/IscU proteins use methionine at position X and prokaryotic proteins do not use methionine at that position.
(XLSX)
