# Supplementary figures and images for: Turning Saccharomyces cerevisiae into a Frataxin-Independent Organism
Source: PLoS Genet. 2015 May 21;11(5):e1005135. doi: 10.1371/journal.pgen.1005135 (PMC4440810; doi:10.1371/journal.pgen.1005135)

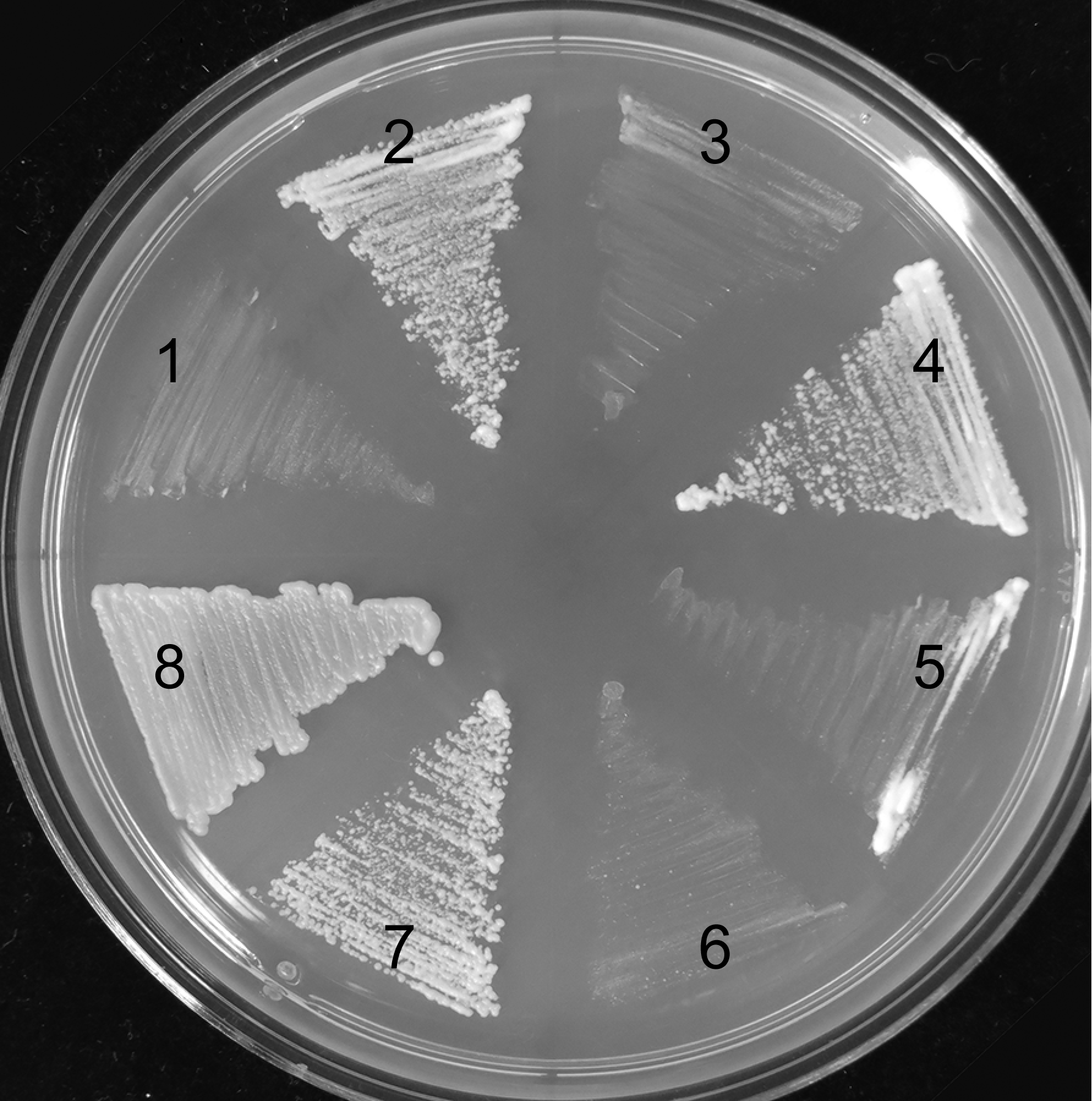

Supplement: S1 Fig — The Δyfh1 shuffle strain 70–31 strain was transformed with ISU2-containing plasmids and control plasmids, and the covering YFH1 plasmid was removed by FOA treatment. The plasmids are: 1) YCplac22-ISU2coding-Met, 2) YCplac22-ISU2coding-Ile 3) YCplac22-ISU2-Met, 4) YCplac22-ISU2-Ile, 5) YCplac22, 6), YCplac22-ISU1-Met, 7) YCplac22-ISU1-Ile, 8) YCplac22-YFH1. Frataxin-bypass was observed for ISU2-Ile and ISU2coding-Ile in which the mutated coding sequence of ISU2 was expressed from the ISU1 promoter. (TIF) [file pgen.1005135.s001.tif]
